# First Time Isolation Report of Bioactive Flavonoids From Water Hyacinth (*Eichhornia crassipes*) Flower Associated With Pharmacological Investigation

**DOI:** 10.1155/bmri/1209329

**Published:** 2026-05-13

**Authors:** Hasin Hasnat, Md. Mirazul Islam, Suriya Akter Shompa, Saima Jahan Riti, Safaet Alam, Tanoy Saha, Mohammad Abdur Rashid

**Affiliations:** ^1^ Department of Pharmacy, School of Pharmaceutical Sciences, State University of Bangladesh, Dhaka, Bangladesh, sub.edu.bd; ^2^ Department of Pharmaceutical Chemistry, Faculty of Pharmacy, University of Dhaka, Dhaka, Bangladesh, du.ac.bd; ^3^ Chemical Research Division, BCSIR Dhaka Laboratories, Bangladesh Council of Scientific and Industrial Research (BCSIR), Dhaka, Bangladesh, bcsir.gov.bd

**Keywords:** anti-inflammatory activity, bioactive flavonoids, cytotoxicity, drug discovery, *Eichhornia crassipes*, molecular docking, phytochemical isolation

## Abstract

**Background:**

*Eichhornia crassipes* (water hyacinth) is an invasive aquatic plant traditionally regarded as an environmental nuisance but increasingly recognized for its phytopharmacological potential.

**Objective:**

This study is aimed at isolating, characterizing, and evaluating the pharmacological activities of bioactive compounds from the dichloromethane (DCM) fraction of the methanolic extract of *E. crassipes* flowers.

**Methods:**

Compounds were isolated through chromatographic techniques and structurally elucidated using ^1^H NMR. Pharmacological activities were assessed using the brine shrimp lethality bioassay for cytotoxicity, egg white–induced edema and human red blood cell membrane stabilization for anti‐inflammatory effects, and oral glucose tolerance tests in mice for hypoglycemic activity. Molecular docking against NF‐*κ*B, TNF‐*α*, EGFR, BCL‐2, GLUT3, and *α*‐amylase, along with ADMET and radar plot analyses, was performed to predict interactions, pharmacokinetics, and drug‐likeness.

**Results and Discussion:**

Five compounds were isolated: 4 ^′^,5,7‐trihydroxyflavone or apigenin (Compound 1), 3 ^′^,4 ^′^,5,7‐tetrahydroxyflavone or luteolin (Compound 2), 5,6,7,8,4 ^′^‐pentahydroxyflavone or nortangeretin (Compound 3), methyl 3,5‐dihydroxy‐4‐methoxybenzoate (Compound 4), and 4‐hydroxybenzoic acid (Compound 5). Both the crude methanolic extract (CME) and the DCM‐soluble fraction (DSF) exhibited strong cytotoxic activity in the brine shrimp lethality assay (LC₅₀ < 2 *μ*g/mL). The DSF further demonstrated potent membrane stabilization (72% protection at 100 *μ*g/mL) and marked hypoglycemic effects. Also, the CME illustrates dose‐dependent anti‐inflammatory activity in the egg white–induced paw edema model (up to 38.96% inhibition at 600 mg/kg) and showed notable hypoglycemic effects in the oral glucose tolerance test in mice. Docking revealed strong binding affinities of flavonoids with key inflammatory and metabolic targets, outperforming reference drugs in several cases. ADMET profiling indicated favorable oral bioavailability, whereas the BOILED‐Egg model confirmed efficient gastrointestinal absorption but limited blood–brain barrier penetration.

**Conclusion:**

This first report of pure compound isolation from *E. crassipes* flowers highlights their multitarget pharmacological potential. The findings suggest that *E. crassipes*, often seen as an ecological burden, represents a sustainable reservoir of bioactive molecules with promising drug discovery applications.

## 1. Introduction

Plants have served as fundamental sources of therapeutic agents for centuries, forming the basis of traditional and modern medicine worldwide [1, 2]. According to the World Health Organization, approximately 80% of the global population relies on traditional medicine, underscoring the enduring significance of plant‐based remedies [2, 3]. Compared to many synthetic drugs, plant‐derived medicines often exhibit broader therapeutic windows and improved tolerability, largely due to their multicomponent and multitarget nature [4, 5]. Traditional healing systems, including Ayurveda, Traditional Chinese Medicine, and Unani medicine, have long utilized various plant parts such as leaves, roots, flowers, and bark for their unique medicinal properties and for the management of a wide range of diseases [1, 2]. The presence of structurally diverse secondary metabolites, particularly flavonoids and phenolic acids, has positioned medicinal plants as valuable reservoirs for lead compound identification and drug development [6, 7]. Their broad acceptability is largely attributed to their generally good safety margins and lower incidence of undesirable side effects [6, 8]. It has been estimated that approximately 25% of modern prescription drugs in developed countries are derived directly or indirectly from plant sources, whereas reliance on herbal medicines may exceed 70%–80% in several Asian and African nations [2, 9]. Even in contemporary medicine, natural products continue to offer promising therapeutic options for complex disorders such as inflammation, oxidative stress, metabolic diseases, and cancer, owing to their accessibility, affordability, and multitarget pharmacological actions. Also their accessibility, affordability, and therapeutic potential make them invaluable to global healthcare [4, 6, 10].

*Eichhornia crassipes*, commonly known as water hyacinth, is an invasive aquatic plant native to the Amazon basin. It has spread globally, thriving in diverse climates and aquatic environments, from lakes to rivers, posing significant ecological challenges [11]. Morphologically, the plant features round to oval leaves with covered petioles and produces blue or lavender flowers. Its air‐filled sacs allow it to float on water surfaces, whereas its extensive root system enables it to absorb nutrients efficiently [12, 13]. Despite its invasive nature, water hyacinth has been utilized in traditional medicine across cultures. In Chinese medicine, it supports spleen health, whereas in the Philippines, it is used as an anti‐inflammatory agent. In Kenya, it aids lactation and menstrual regulation, and its beans are consumed to address digestive issues. Modern research has revealed its potential anticancer properties, alongside its antifungal, antibacterial, and wound‐healing capabilities [10, 14].

Water hyacinth belongs to the Pontederiaceae family and is rich in bioactive compounds such as alkaloids, flavonoids, phenols, and terpenoids [15, 16]. These compounds contribute to its neuropharmacological effects, including analgesic, anti‐epileptic, and memory‐enhancing properties [10, 17]. Additionally, it exhibits hepatoprotective, antioxidant, and antitumor activities, making it a versatile resource for pharmaceutical and cosmetic applications [18, 19]. Its ability to absorb heavy metals further highlights its utility in phytoremediation. The plant′s diverse phytochemical composition supports its use in treating gastrointestinal disorders, inflammation, and pain, solidifying its role in both traditional and modern medicine [20, 21].

Chronic conditions like diabetes mellitus (DM) remain a significant global health challenge, affecting millions of people worldwide [22]. Given the limitations and side effects of existing antidiabetic medications, researchers are exploring alternative treatments. Medicinal plants have gained attention as a complementary therapy, potentially offering fewer adverse effects [23, 24]. Meanwhile, gastrointestinal disorders particularly diarrhea remain a major global public health concern, disproportionately affecting populations in developing countries and contributing significantly to morbidity and mortality, especially among children and immunocompromised individuals [25, 26]. In this context, traditional herbal medicine has gained increasing scientific attention as a complementary and accessible therapeutic approach. Endorsed by the World Health Organization, plant‐based remedies play an important role in primary healthcare by offering culturally accepted, affordable, and locally available interventions, thereby effectively bridging traditional knowledge with modern medical systems [3, 27].

In the field of cancer research, advancements over the past two decades have transformed treatment strategies through the identification of shared molecular mechanisms across multiple tumor types [27, 28]. However, chemotherapy‐induced neurotoxicity remains a significant clinical challenge, often resulting in long‐term neurological complications and reduced quality of life among cancer patients [29]. This limitation underscores the need for safer and more effective therapeutic approaches, where natural products and plant‐derived compounds may serve as promising alternatives due to their multitarget activity and comparatively favorable safety profiles [4, 30].

Chronic inflammation‐induced pain, mediated by excessive production of prostaglandins, cytokines, and reactive oxygen species, is commonly managed using nonsteroidal anti‐inflammatory drugs, opioids, and nonopioid analgesics [31]. Although these agents provide symptomatic relief, prolonged use is frequently associated with adverse effects including gastrointestinal toxicity, cardiovascular risk, tolerance, and organ damage [32]. Consequently, increasing scientific attention has been directed toward safer therapeutic alternatives, particularly secondary metabolites derived from medicinal plants, which have demonstrated significant anti‐inflammatory and analgesic activities through modulation of COX enzymes, NF‐*κ*B signaling, and cytokine expression [33, 34].

This study is aimed at exploring the phytochemical composition of *E. crassipes* flower extracts using advanced chromatographic and spectroscopic techniques. By integrating in vivo, in vitro, and in silico approaches, the research evaluated the anti‐inflammatory, hypoglycemic, and cytotoxic effects of the flower extract. To address the challenge of correlating phytochemical identity with biological activity, an in silico approach was employed to predict interactions between the identified compounds and relevant biological targets, providing preliminary insights into their therapeutic mechanisms. This comprehensive methodology, combining experimental and computational analyses, offers a thorough assessment of the extract′s potential, bridging the gap between chemical composition and biological efficacy, and highlighting the therapeutic promise of *E. crassipes* flowers in addressing inflammation, hyperglycemia, and cytotoxicity.

## 2. Materials and Method

### 2.1. Collection of Plant Material

The flowers of *E. crassipes* (Mart.) Solms were collected from the Hawor area of Austagram, Kishoreganj, Dhaka, Bangladesh, on November 11, 2022. The plant material was authenticated by Khondokar Kamrul Islam, Senior Scientific Officer, at the Bangladesh National Herbarium on December 6, 2022, and assigned the unique accession number DACB 87254. The collected samples were processed and preserved in the phytochemical research laboratory of the School of Pharmacy at the State University of Bangladesh for further analysis.

### 2.2. Plant Extraction

The flowers of *E. crassipes* were carefully washed and dried in shades until the moisture content reached approximately 10%. The dried plant material was then ground into a coarse powder using a grinding apparatus. Next, 800 g of the dried flower powder was placed into a 5‐L round‐bottomed flask, and 2.5 L of methanol (analytical grade, ≥ 96% purity) was added. The mixture was allowed to macerate for 21 days under controlled laboratory conditions in a dark and cool environment with periodic stirring to facilitate solvent penetration and diffusion of phytochemical constituents, a procedure consistent with maceration‐based extraction methods commonly employed in phytochemical investigations of medicinal plants [35–38]. After this period, the mixture (32% w/v) was filtered using Whatman No. 1 filter paper and a fresh cotton plug. This extraction process was repeated three times using analytical‐grade methanol. The filtrate was then concentrated using a Buchi Rotavapor under low temperature and pressure, yielding 83.5 g of crude extract (10.43% yield).

For further separation, the Kupchan fractionation method was applied to the crude methanolic extract (CME). This technique, initially described by VanWagenen et al. (1993), involves sequential solvent–solvent partitioning using solvents of increasing polarity [39]. In this study, the crude extract (50.0 g) was fractionated using petroleum ether (PET), dichloromethane (DCM), ethyl acetate (EA), and distilled water. Each solvent selectively extracted compounds based on their polarity: PET for nonpolar compounds, DCM for slightly polar compounds, EA for moderately polar compounds, and water for highly polar compounds. After fractionation, each solvent phase was concentrated using rotary evaporation, yielding the following fractions: PET‐soluble fraction (PSF, 16.1 g), DCM‐soluble fraction (DSF, 11.12 g), EA‐soluble fraction (ESF, 12.18 g), and aqueous‐soluble fraction (ASF, 7.32 g). This method demonstrates a systematic and efficient approach to isolating diverse compound classes.

### 2.3. Isolation of Compounds

The DSF of the flower of *E. crassipes* was subjected to gel permeation chromatography (GPC/SEC) conducted using Sephadex LH‐20 (Sigma‐Aldrich), whereas preparative thin‐layer chromatography (PTLC) and thin‐layer chromatography (TLC) were performed on silica gel 60 F254 aluminum sheets (Merck, Germany) with a thickness of 0.25 mm. The TLC plates were visualized under a UV lamp (UVGL‐58, United States) at wavelengths of 254 and 365 nm. For further visualization, the plates were sprayed with a vanillin–sulfuric acid mixture and heated at 100°C for 5 min. Pure compounds were isolated using the PTLC method, and their purity was confirmed through subsequent spot TLC analysis. The isolated compounds were then analyzed using ^1^H NMR spectroscopy. The ^1^H NMR spectra were recorded on a Bruker 600‐MHz spectrometer using CDCl_3_ or MeOD as solvents. Chemical shifts were reported in *δ* (ppm) relative to tetramethylsilane (TMS) as the internal reference [40].

### 2.4. In Vitro Activities

#### 2.4.1. Cytotoxic Activity

The cytotoxicity of various extracts from *E. crassipes* was evaluated using the brine shrimp lethality bioassay (*Artemia salina*), with vincristine sulfate serving as the reference anticancer compound [41, 42]. A simulated saltwater solution was prepared by dissolving 38 g of NaCl in 1000 mL of distilled water, followed by adjusting the pH to 8.0 using NaOH. Each of the four fractionated extracts was dissolved in dimethyl sulfoxide at a concentration of 50 *μ*L/5 mL to prepare the test solutions in simulated seawater. Serial dilutions were performed for both the fractionated extracts and the vincristine standard solution, starting from 400 to 0.78125 *μ*g/mL for the extracts and from 20 to 0.039 *μ*g/mL for the standard. Each dilution step reduced the concentration by half. Ten live brine shrimp nauplii were placed in each test tube and maintained at room temperature (25^°^C ± 1^°^C). After 24 h, the number of dead nauplii was recorded. The mortality percentage was calculated using the formula

% of mortality = (*N*1/*N*0) × 100*%*

where *N*_0_ represents the total number of nauplii used, and *N*_1_ represents the number of dead nauplii.

#### 2.4.2. Membrane‐Stabilizing Activity

The membrane‐stabilizing activity of the plant samples was evaluated using hemolysis [43]. Human blood samples were obtained from healthy adult volunteers who were members of the research team. Ethical approval for the involvement of human participants was granted by the Institutional Ethical Review Committee of the State University of Bangladesh under Approval Number 2023‐10‐30/SUB/I‐ERC/003. Written informed consent was obtained from the healthy adult volunteer whose blood sample was used in the membrane‐stabilizing assay. Blood samples were collected using ethylenediaminetetraacetic acid (EDTA) as an anticoagulant. The collected blood was washed with isotonic saline solution (0.9% NaCl) and centrifuged at 3000 rpm for 10 min. This washing step was repeated three times, after which a stock erythrocyte suspension was prepared for subsequent analyses.

##### 2.4.2.1. Hemolysis Induced by a Hypotonic Solution.

A hypotonic solution was utilized for this experiment. Test materials, including CME, PSF, DSF, ESF, and ASF at a concentration of 2.0 mg/mL, along with aspirin (standard) at 0.10 mg/mL, were separately mixed with 0.5 mL of stock erythrocyte suspension, 4.5 mL of hypotonic solution (0.3% NaCl), and 10 mM sodium phosphate buffer (pH 7.4). For the negative control group, water was used instead of the test material. The mixtures were incubated at room temperature for 10 min, followed by centrifugation at 3000 rpm for 10 min. The absorbance (optical density, OD) of the supernatant was measured at 540 nm using a UV spectrophotometer.

The percentage of hemolysis was calculated using the formula

% hemolysis = (1 − OD_2_/OD_1_) × 100*%*.

Here, OD₁ represents the OD of the hypotonic‐buffered saline solution alone (control group), and OD₂ represents the OD of the test sample in the hypotonic solution.

##### 2.4.2.2. Hemolysis Induced by Heat.

Initially, 4.5 mL of isotonic solution and 10 mM sodium phosphate buffer were added to two sets of centrifuge tubes, along with either aspirin (0.10 mg/mL) or the test samples (2.0 mg/mL). Then, 30 *μ*L of stock erythrocytes was added to each tube. One set of tubes was incubated in a water bath at 54°C for 20 min, whereas the other set was kept in an ice bath at 0°C–5°C for the same duration. After incubation, the mixtures were centrifuged at 3000 rpm for 3 min, and the absorbance was measured at 540 nm using a UV spectrophotometer.

The percentage of hemolysis was calculated using the formula

% hemolysis= (OD_2_ − OD_1_/OD_3_ − OD_1_) × 100*%*.

Here, OD₁ represents the unheated test sample, OD₂ represents the heated test sample, and OD_3_ represents the heated control sample.

### 2.5. In Vivo Activities

#### 2.5.1. Animal Preparation

Swiss white mice of both sexes, aged 4–5 weeks, were obtained from the Animal Resource Branch of the International Centre for Diarrheal Diseases and Research, Bangladesh (ICDDR, B), for the in vivo study. The mice were housed in polypropylene cages under controlled conditions, including a 12‐h light–dark cycle, a temperature of 24^°^C ± 2^°^C, and a relative humidity of 60%–70%. They were provided with ICDDR, B‐formulated rodent food and water ad libitum. To ensure acclimatization to the experimental environment and reduce sensitivity to environmental changes, the mice were kept under these conditions for at least 4 days prior to the study. All experiments complied with ethical guidelines and regulations for the use and care of laboratory animals. The study followed the approved Animal Use Protocol from the Ethics Committee, adhering to the guidelines of the US National Institutes of Health for the Care and Use of Laboratory Animals. Additionally, recommendations from the Federation of European Laboratory Animal Science Associations (FELASA) were implemented to minimize pain and stress in the animals. The study was approved by the Animal Ethics Committee of the State University of Bangladesh, Dhaka (2023‐10‐30/SUB/I‐ERC/005).

Animals were routinely monitored throughout the experimental period for any signs of pain or distress, including but not limited to a reduction in body weight exceeding 20%, marked lethargy, impaired mobility, difficulty accessing food or water, lack of responsiveness to external stimuli, sustained piloerection, abnormal posture, respiratory difficulty, or seizure‐like activity. However, none of these indicators were observed at any stage of the study, as all experimental procedures were noninvasive and did not cause measurable discomfort or harm to the animals. All personnel involved in animal handling and monitoring had received appropriate training in laboratory animal husbandry, recognition of distress symptoms, and adherence to institutional animal care guidelines. Following completion of the experimental phase, all animals were continuously observed twice daily for an additional 2‐month postexperimental period. During this follow‐up, no abnormal clinical signs, behavioral changes, or health‐related complications were detected. At the end of the observation period, all animals were returned to standard housing conditions and maintained under routine care without any adverse outcomes.

#### 2.5.2. Acute Oral Toxicity Test

Mice were administered high oral doses of 2000 and 4000 mg/kg of the methanol‐soluble crude extract of *E. crassipes* (CME), under standard laboratory conditions, following the fixed‐dose method outlined in the OECD Guidelines 420 [44]. Over a 72‐h observation period postadministration, no signs of allergic reactions, behavioral changes (such as sedation or excitability), or mortality were observed. Based on these findings, the doses of 200, 400, and 600 mg/kg (body weight, orally) were selected as safe for further in vivo activity studies.

#### 2.5.3. Anti‐Inflammatory Activity

The anti‐inflammatory effect of crude methanol extract of the flower of *E. crassipes* was assessed by the egg white (egg albumin)–induced paw edema in mice model [45, 46]. After an overnight fast, 24 rats were divided into five groups (*n* = 4 per group) and treated intraperitoneally as follows: Group I (negative control) received only the water orally, Group II was administered diclofenac sodium orally at a dose of 10 mg/kg body weight, and Groups III–V (treatment groups) received CME at doses of 200, 400, and 600 mg/kg body weight, respectively. After 30 min of treatment, inflammation was induced by injecting 0.1 mL of fresh hen′s egg white (egg albumin) subcutaneously into the subplantar region of the right hind paw of each mice in all groups. Paw thickness was measured using a digital vernier caliper immediately before (0 min) and at 30, 60, 120, and 240 min after egg albumin injection. The severity of edema was assessed by calculating the mean increase in paw thickness (millimeters), determined as the difference between paw thickness at 0 h and at each subsequent time point. The anti‐inflammatory effect of the tested compounds was expressed as the percentage inhibition of paw edema, calculated using the formula

Percentage inhibition (%) = (1 − dc/dt) × 100*%*

where dt represents the mean difference in paw thickness of treated animals after egg albumin injection, and dc is the mean difference in paw thickness of control animals after egg albumin injection.

#### 2.5.4. Hypoglycemic Activity

The hypoglycemic effect of the methanolic extract of *E. crassipes* flowers was evaluated using the oral glucose tolerance test in mice [27]. Five groups of animals containing four mice in each serve as one positive control, one negative control, and three treatment groups. During the experiment, test samples at doses of 200, 400, and 600 mg/kg were administered orally to the treatment groups of mice. Glibenclamide (10 mg/kg body weight) was used as the reference drug and administrated orally as positive control, whereas the negative control group was given only water. After 30 min, all mice were given a 10% glucose solution. Blood glucose levels were measured at 0 min and then at 30, 60, and 120 min using a glucometer. The percentage reduction in blood glucose levels was calculated using the following formula:

% reduction in blood glucose = (BGcontrol − BGtest/BGcontrol) × 100*%*.

Here, BG_control_ represents the average blood glucose level of the negative control group, and BG_test_ represents the average blood glucose level of the test groups or positive control group.

### 2.6. Statistical Analysis

Statistical analysis of in vivo studies was performed using GraphPad Prism 5.2 (GraphPad Software Inc., La Jolla, California, United States), with data presented as mean ± standard error of the mean (SEM). To assess statistical significance, one‐way analysis of variance (ANOVA) followed by Dunnett′s test was utilized. The levels of significance were denoted as ∗p < 0.05, ∗∗p < 0.01, and ∗∗∗p < 0.001 [10, 47].

### 2.7. Docking

#### 2.7.1. Software

The isolated and identified compounds from DSF of *E. crassipes* flower were subjected to computational docking studies using widely recognized software tools, such as PyRx, PyMoL 2.3, Discovery Studio 4.5, and Swiss PDB viewer [48].

#### 2.7.2. Ligand Preparation

For ligand preparation, identified compounds were searched in the PubChem database (https://pubchem.ncbi.nlm.nih.gov/), and their 3D structures were downloaded in SDF format. Additionally, the 3D structures of standard reference compounds, aspirin, glibenclamide, vincristine, and lapatinib, were also obtained. These ligands, along with their respective PubChem CIDs, were imported into Discovery Studio 4.5. The PM6 semi‐empirical quantum mechanical method was subsequently applied for geometry optimization of all phytochemicals, thereby improving structural reliability and docking accuracy [49, 50].

#### 2.7.3. Receptor Preparation

For receptor preparation, the 3D crystal structures of target proteins listed on Table 1 were retrieved from the Protein Data Bank (RCSB PDB, https://www.rcsb.org) [58]. These protein structures were processed in PyMOL 2.3 to remove water molecules and ligands/residues. Nonpolar hydrogen atoms were added, and energy minimization was performed using Swiss PDB viewer [48]. Ligand–receptor binding analysis was performed using PyRx integrated with AutoDock Vina, employing a semiflexible docking approach in which ligands were flexible whereas receptor structures remained rigid. Target proteins were prepared as macromolecules, and active‐site amino acid residues were selected based on previously reported literature to ensure accurate and biologically relevant ligand binding within the target sites [59, 60].

**Table 1:** Selection of target site and grid mapping of target receptors for molecular docking of isolated compounds from the DCM fraction of the methanolic extract of *E. crassipes* flower.

Target activity	Receptor	Standard	Target binding sites	Reference	Grid box
Anti‐inflammatory	Nuclear factor‐kappa B (NF‐*κ*B) [PDB ID: 5LDE]	Aspirin (PubChem ID: 2244)	ALA3, LYS4, PRO5, GLU6, GLY7, SER23, VAL26, ILE27, PHE30, GLN31, LEU34, ASP39, GLY40, LEU41, GLU42, VAL43, TYR44, GLU45, ASN56	[51]	Center *x* = −13.2764015647, *y* = 10.6995882594, *z* = −5.26208942928
					Dimension *x* = 22.3973098127, *y* = 25.0472976911, *z* = 26.8480453359
	Tumor necrosis factor alpha (TNF‐*α*) [PDB ID:2az5]		A chain‐ TYR59, TYR119, LEU 120, GLY121, TYR151; B chain‐ TYR59, SER60, GLN61, TYR 119, LEU120, GLY121	[8, 52]	Center *x* = −19.8024359334, *y* = 74.370137214, *z* = 37.7543263647
					Dimension *x* = 19.7708837032, *y* = 23.0264797793, *z* = 15.8434154561
Cytotoxic	Epidermal growth factor receptor (EGFR) [PDB ID: 1XKK]	Vincristine (PubChem ID: 5978) and lapatinib (PubChem ID: 208908)	ASP800, TYR998, LEU1001, CYS797, GLY719, GLY796, LEU718, VAL726, LYS745, ASP855, THR854, PHE997, GLU804, TYR801, PHE795, SER720, GLY721, THR790, LEU777, CYS775, PHE856, MET766, LEU788, LEU758, LEU844, ASN842	[53]	Center *x* = 16.006586689, *y* = 34.5683228338, *z* = 35.8171547412
					Dimension *x* = 25.555877042, *y* = 20.2918178718, *z* = 32.6048010387
	B‐cell lymphoma 2 (BCL‐2) [PDB ID: 2W3L]		A chain‐ PHE63, TYR67, PHE71, MET74, ARG88, VAL92, GLU95, LEU96, ARG105, ALA108	[41, 54]	Center *x* = 41.2226620516, *y* = 27.7548039658, *z* = −12.4383788994
					Dimension *x* = 18.5200959249, *y* = 22.955075927, *z* = 21.4758180396
Hypoglycemic	Glucose transporter Type 3 (GLUT3) [PDB ID: 4ZWB]	Glibenclamide (PubChem ID: 3488)	TYR26, THR28, GLY29, VAL30, LEU167, THR191, PRO194, GLN198, ILE309, GLY312, VAL313, THR347, TRP410, LEU418, PHE442	[55, 56]	Center *x* = 106.621953547, *y* = 10.3865389227, *z* = 60.6138207213
					Dimension *x* = 45.5316986867, *y* = 25.2859512382, *z* = 33.779602352
	*α*‐Amylase [PDB ID: 1HNY]		TRP59, TRY151, LEU162, THR163, ALA198, LYS200, GLU233, ASP300, HIS305	[57]	Center *x* = 9.22188284497, *y* = 43.5404159291, *z* = 21.1508663996
					Dimension *x* = 23.4338343101, *y* = 19.0034466204, *z* = 19.8926565284

#### 2.7.4. ADME/T

In modern drug development, computational approaches focusing on pharmacokinetics—absorption, distribution, metabolism, excretion, and toxicity (ADMET)—have become essential for evaluating drug‐like properties and bioavailability. These analyses are critical tools for understanding the pharmacological profile of potential drug candidates (https://biosig.unimelb.edu.au/pkcsm/prediction). Additionally, the Swiss ADME online platform (https://www.sib.swiss) was utilized to predict drug‐likeness based on Lipinski′s rules and pharmacokinetic parameters. According to Lipinski′s criteria, a compound is considered suitable for oral administration if it meets the following conditions: a molecular weight of less than 500 atomic mass units (amu), no more than five hydrogen bond donors, no more than 10 hydrogen bond acceptors, and a lipophilicity value (LogP) of 5 or less [36].

## 3. Result

### 3.1. Phytochemical Analysis

**Figure 1:**
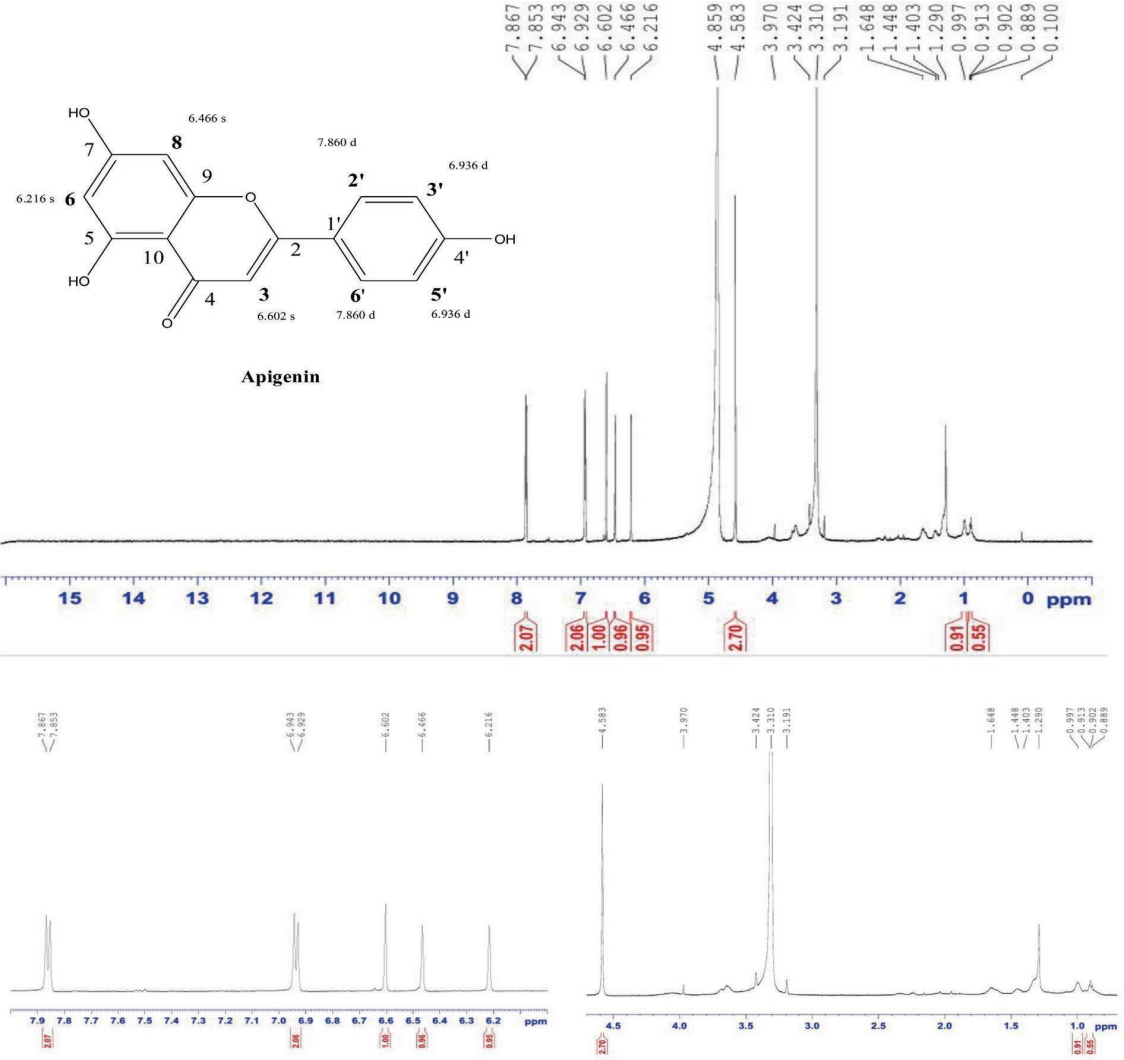
^1^H NMR spectrum of 4 ^′^,5,7‐trihydroxyflavone (apigenin) isolated from the DCM fraction of the methanolic extract of the flower of *E. crassipes*.

Compound 1 was a yellowish powder (5.2 mg), Rf = 0.85 (85% EA, 15% toluene); the obtained ^1^H NMR spectra showed consistency with the previously reported data presented in Table 2. The ^1^H NMR spectrum (MeOD, 600 MHz) showed peak *δ* 7.86 (2H, *d* = 8.4 Hz, H‐2 ^′^, H‐6 ^′^), *δ* 6.936 (2H, *d* = 8.4 Hz, H‐3 ^′^, H‐5 ^′^), *δ* 6.602 (1H, s, H‐3), *δ* 6.466 (1H, s, H‐8), and *δ* 6.466 (1H, s, H‐6) (Figure 1).

**Table 2:** The ^1^H NMR of the isolated compounds compared with reference spectroscopic data.

Serial	Compound name	Position	^1^H NMR values (*δ*in ppm)	Reference values (*δ*in ppm)	Reference
			MeOD, 600 MHz	Pyridine‐d5, 400 MHz	
1	Apigenin	H‐2 ^′^, H‐6 ^′^	7.86 (*d*, 8.4, 2H)	7.99 (2H, *d*, *J* = 8.7 Hz)	[61]
		H‐3 ^′^, H‐5 ^′^	6.94 (*d*, 8.4, 2H)	7.08 (2H, *d*, *J* = 8.7 Hz)	
		H‐3	6.60 (s, 1H)	6.78 (1H, s)	
		H‐8	6.47 (s, 1H)	6.69 (1H, *d*, *J* = 2.0 Hz)	
		H‐6	6.21 (s, 1H)	6.69 (1H, *d*, *J* = 2.0 Hz)	
			MeOD‐d4, 600 MHz	CD3OD, 500 MHz	
2	Luteolin	H‐6	6.21 (s, 1H)	6.19 (1H, *d*, *J* = 2.0 Hz)	[62]
		H‐8	6.45 (s, 1H)	6.42 (1H, *d*, *J* = 2.0 Hz)	
		H‐3	6.55 (s, 1H)	6.52 (1H, s)	
		H‐5 ^′^	6.91 (*d*, 7.8, 1H)	6.88 (1H, *d*, *J* = 8.5 Hz)	
		H‐2 ^′^, H‐6 ^′^	7.38 (*d*, 2H)	7.36 (2H, m)	
			CDCl_3_ at 600 MHz	400 MHz, CDCl3	
3	5,6,7,8,4 ^′^‐Pentahydroxyflavone (nortangeretin)	H‐1 ^′^	7.35 (*d*, *J* = 8.4 Hz)	7.31 (*d*, *J* = 8.4 Hz)	[63]
		H‐5 ^′^	6.89 (*d*, *J* = 8.4 Hz)	6.86 (*d*, *J* = 7.6 Hz)	
		H‐5	12.56 (s)	12.08 (*d*, *J* = 6.0)	
			7.43 (s)		
			7.08 (s)		
			CDCl_3_, 600 MHz	CD3OD, 500 MHz	
4	Methyl 3,5‐dihydroxy‐4‐methoxybenzoate	H‐2	7.23 (s, 2H)	7.02 (2H,s)	[64]
		H‐6	7.23 (s, 2H)	7.02 (2H,s)	
			3.87 (s, –OCOCH3)	3.83 (s, –OCOCH3)	
			3.89 (s, –OCH3)	3.86 (s, –OCH3)	
			CDCl_3_, 600 MHz	Me‐D4, 600 MHz	
5	4‐Hydroxybenzoic acid	H‐2, H‐3	7.99 (*d*, 7.8)	7.87 (*d*, 8.7)	[65]
		H‐3, H‐5	6.88 (*d*, 8.4)	6.81 (*d*, 8.7)	

**Figure 2:**
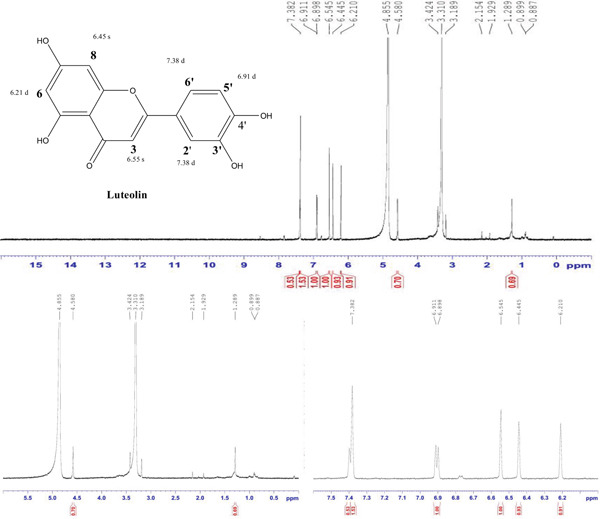
^1^H NMR spectrum of 3 ^′^,4 ^′^,5,7‐tetrahydroxyflavone (luteolin) isolated from the DCM fraction of the methanolic extract of the flower of *E. crassipes*.

Compound 2 was a light yellow powder (3.4 mg), Rf = 0.63 (80% EA, 20% toluene); ^1^H NMR signals revealed close agreement with earlier published values summarized in Table 2. ^1^H NMR (MeOD‐d4, 600 MHz) showed peak at *δ* 6.210 (1H, s, H‐6), *δ* 6.445 (1H, s, H‐8), *δ* 6.545 (1H, s, H‐3), *δ* 6.905 (1H, *d*, *J* = 7.8 Hz, H‐5 ^′^), and *δ* 7.382 (2H, d, H‐2 ^′^, H‐6 ^′^) (Figure 2).

**Figure 3:**
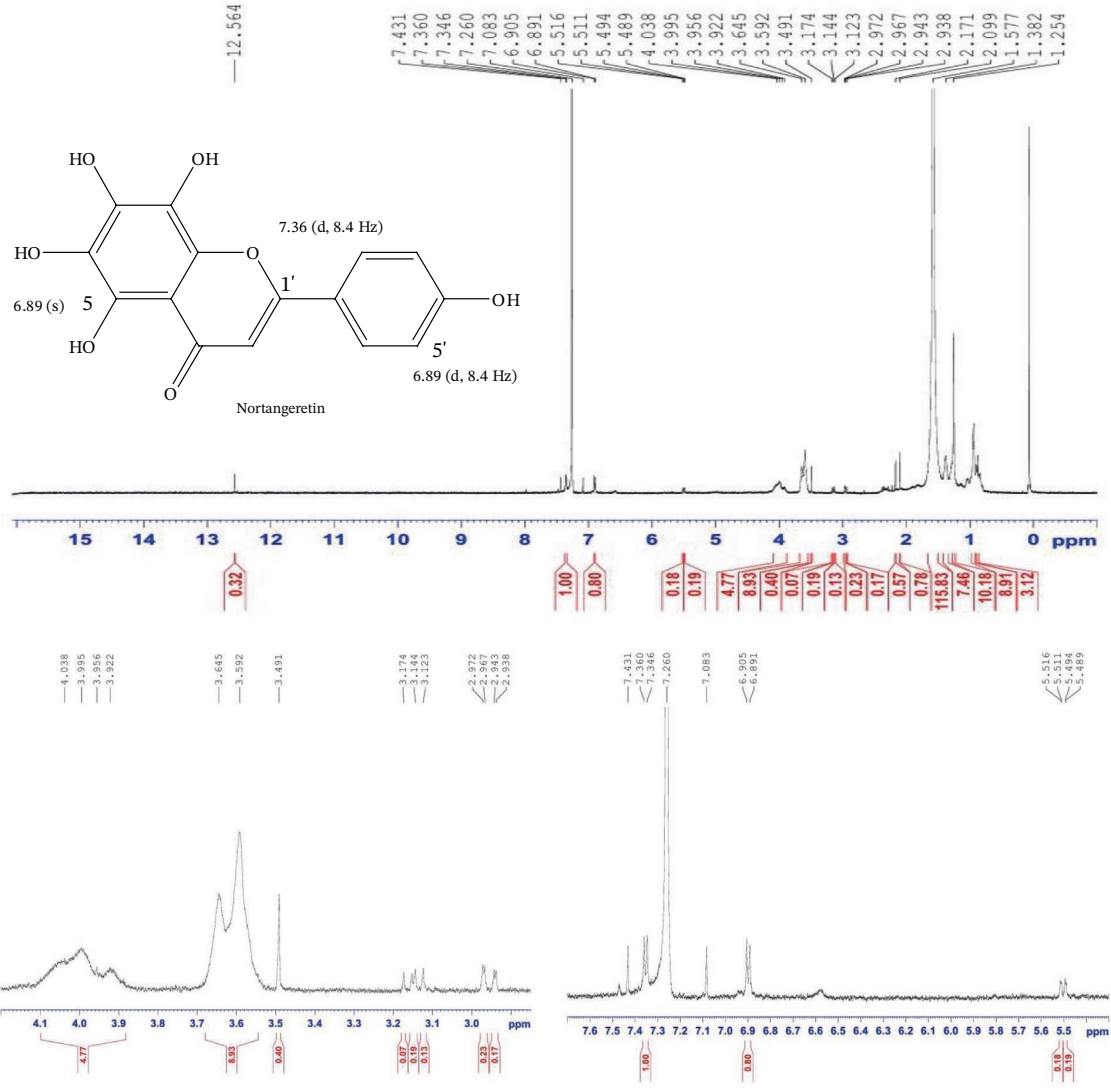
^1^H NMR spectrum of 5,6,7,8,4 ^′^‐pentahydroxyflavone (nortangeretin) isolated from the DCM fraction of the methanolic extract of the flower of *E. crassipes*.

Compound 3 was a colorless crystal (4.2 mg), Rf = 0.6 (50% EA, 50% toluene); ^1^H NMR data were found similar to the previously published data in Table 2. The ^1^H NMR spectrum (CDCl_3_, 600 MHz) displays signals consistent with a flavanone structure. The spectrum shows 12.564 (s, 1H), 7.431 (s, 1H), 7.346 (*d*, *J* = 8.4 Hz, 1H), 7.083 (s, 1H), 6.891 (*d*, *J* = 8.4 Hz, 1H), 5.489–5.516 (*dd*, *J* = 13.2, 10.2 Hz, 1H), 2.938–2.972 (*dd*, *J* = 3.0, 14.4 Hz, 1H), and 3.123–3.174 (*dd*, *J* = 12.6, 18.0 Hz, 1H) (Figure 3).

**Figure 4:**
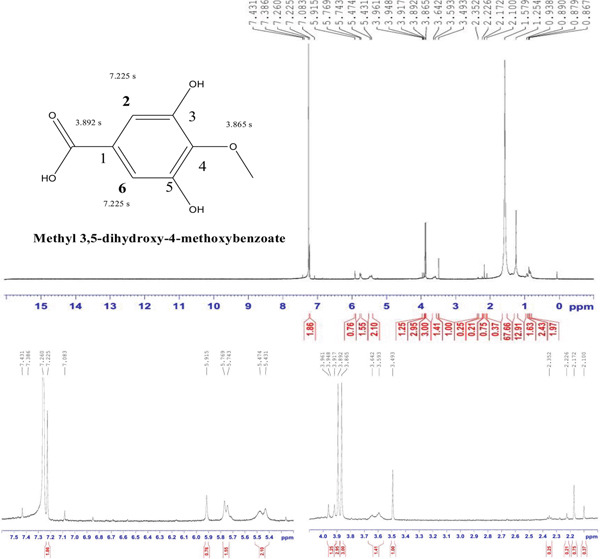
^1^H NMR spectrum of methyl 3,5‐dihydroxy‐4‐methoxybenzoate isolated from the DCM fraction of the methanolic extract of the flower of *E. crassipes*.

Compound 4 was a yellowish solid powder (3.9 mg), Rf = 0.71 (80% EA, 20% toluene); ^1^H NMR results aligned well with the reference data documented in Table 2. The ^1^H NMR spectrum (CDCL3, 600 MHz) illustrates the signals at *δ* 7.225 (2H, s, H‐2), *δ* 7.225 (2H, s, H‐6), *δ* 3.865 (s, –OCOCH3), and *δ* 3.892 (s, –OCH3) (Figure 4).

**Figure 5:**
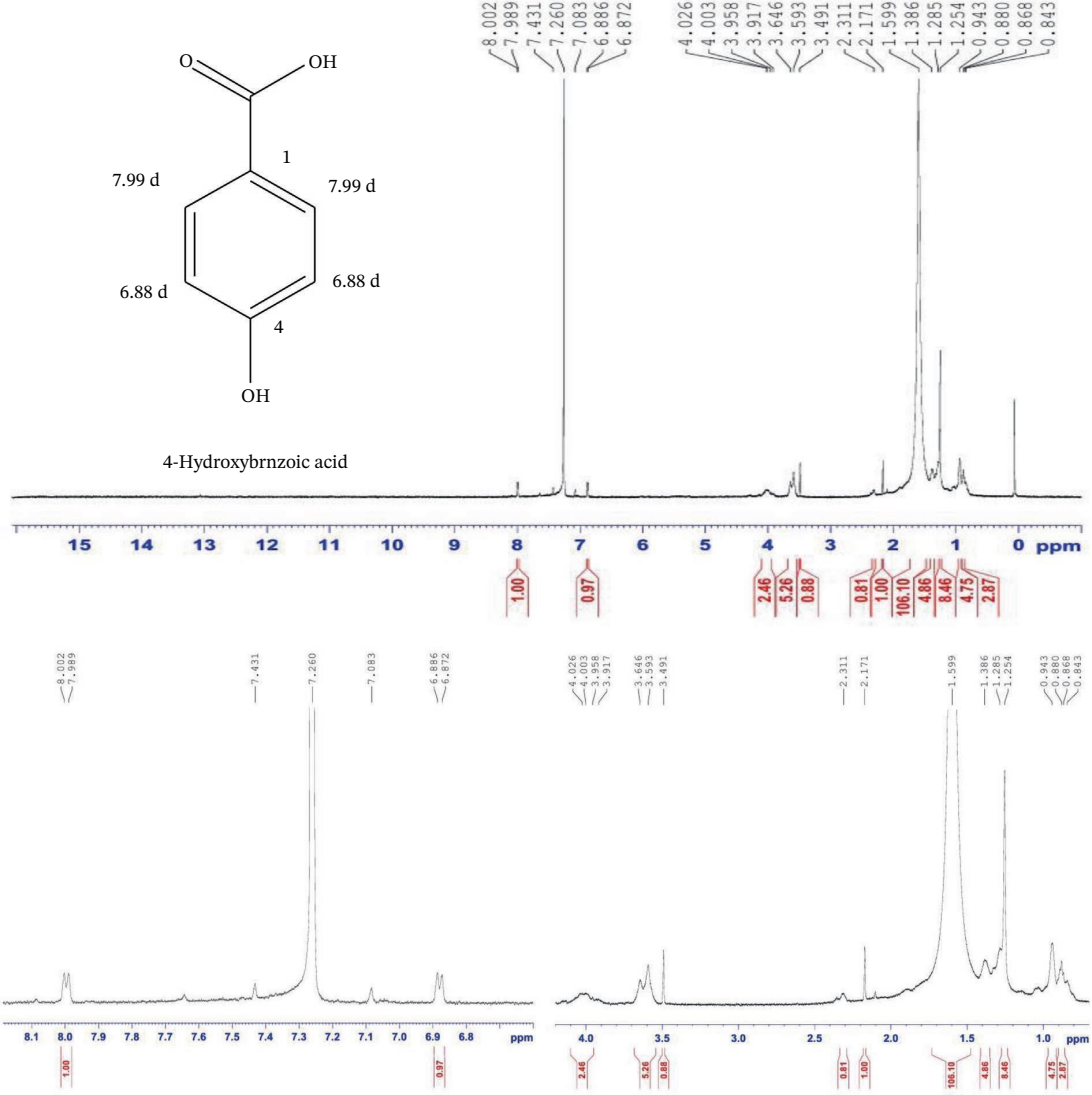
^1^H NMR spectrum of 4‐hydroxybenzoic acid isolated from the DCM fraction of the methanolic extract of the flower of *E. crassipes*.

Compound 5 was off‐white solid powder (3.3 mg), Rf = 0.43 (80% EA, 20% toluene); comparison of ^1^H NMR findings indicated strong similarity to those previously reported in Table 2. The ^1^H NMR (CDCL3, 600 MHz) showed peak at *δ* 7.99 (2H, *d*, *J* = 7.8 Hz, H‐2,3) and *δ* 6.88 (2H, *d*, *J* = 8.4 Hz, H‐3,5) (Figure 5).

### 3.2. Cytotoxic

**Figure 6:**
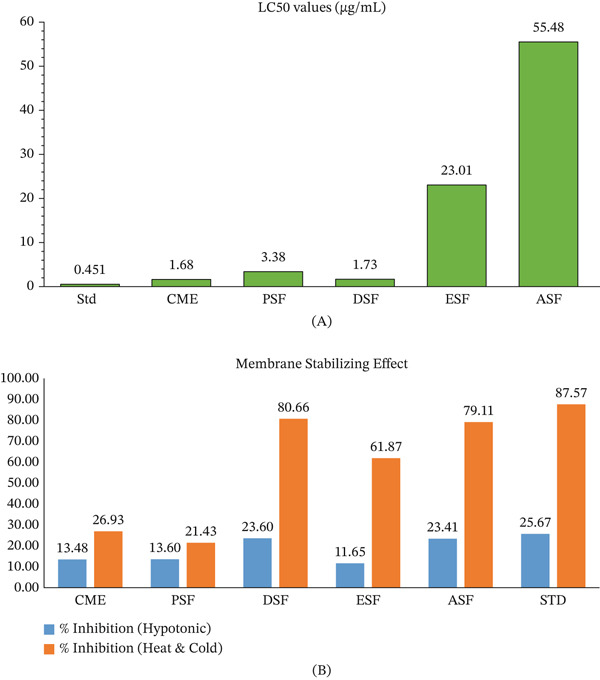
Cytotoxic and membrane‐stabilizing activities of different fractions of the methanolic extract of the flower of *E. crassipes*, where A denoted cytotoxic activities and B denoted membrane‐stabilizing activities.

The cytotoxic effects of different extracts were assessed via a brine shrimp lethality bioassay, and LC₅₀ values were calculated using regression analysis (Figure 6A). The standard compound showed a strong cytotoxic profile with an LC₅₀ of 0.451 *μ*g/mL, indicating excellent model fit. Among the extracts, CME exhibited the most potent cytotoxic effect with an LC₅₀ of 1.68 *μ*g/mL, followed by DSF (1.73 *μ*g/mL) and PSF (3.38 *μ*g/mL), showing moderate toxicity. ESF (23.01 *μ*g/mL) and ASF (55.48 *μ*g/mL) had the highest LC₅₀ values, indicating weak or negligible cytotoxicity.

### 3.3. Membrane Stabilizing

The membrane stabilization assay revealed significant protective effects (Figure 6B). Under hypotonic‐induced hemolysis, DSF (23.60%) and ASF (23.41%) were the most active, whereas CME and PSF showed moderate stabilization. In the heat/cold‐induced model, both DSF (80.66%) and ASF (79.11%) exhibited strong stabilization, nearly comparable to the standard diclofenac sodium (87.57%). This suggests that the bioactives in DSF and ASF may play important roles in protecting membranes under stress conditions.

### 3.4. Anti‐inflammatory

The CME of *E. crassipes* flowers demonstrated significant, dose‐dependent anti‐inflammatory activity in the egg white–induced paw edema assay (Table 3). In the control group, edema progressively increased from 6.53 ± 0.31 mm at baseline to 10.61 ± 0.17 mm at 240 min. Treatment with CME at 200, 400, and 600 mg/kg produced progressive inhibition of inflammation, with effects becoming more pronounced over time. At 200 mg/kg, CME showed 6.02% inhibition at 30 min, rising to 33.82% at 240 min (*p* < 0.001). The 400 mg/kg dose exhibited 4.89% inhibition at 30 min and reached 34.67% at 240 min (*p* < 0.001). The highest dose, 600 mg/kg, demonstrated the strongest effects, with inhibition increasing from 5.70% at 30 min to 38.96% at 240 min (*p* < 0.001). Notably, while the standard (STD) drug exerted earlier significant effects (30–90 min), all CME doses displayed sustained inhibition at later time points (120–240 min), in several cases surpassing the standard. These findings suggest that *E. crassipes* CME exerts prolonged anti‐inflammatory effects, potentially mediated through suppression of prostaglandin synthesis or modulation of pro‐inflammatory cytokines.

**Table 3:** Anti‐inflammatory activities of the methanolic crude extract of the flower of *E. crassipes* through egg white–induced paw edema assay.

Group	Average thickness					% inhibition of paw edema			
	0 min	30 min	60 min	120 min	240 min	30 min	60 min	120 min	240 min
Control	6.53 ± 0.31	8.43 ± 0.17	9.20 ± 0.19	9.61 ± 0.22	10.61 ± 0.17	—	—	—	—
CME 200	7.54 ± 0.28	7.92 ± 0.18	8.25 ± 0.15 ∗∗	7.48 ± 0.26 ∗∗∗	7.02 ± 0.18 ∗∗∗	6.02	10.27	22.15	33.82
CME 400	7.51 ± 0.21∗	8.02 ± 0.24	7.42 ± 0.18 ∗∗∗	6.93 ± 0.14 ∗∗∗	6.93 ± 0.09 ∗∗∗	4.89	19.38	27.82	34.67
CME 600	7.41 ± 0.22	7.95 ± 0.18	7.21 ± 0.12 ∗∗∗	6.88 ± 0.05 ∗∗∗	6.48 ± 0.10 ∗∗∗	5.70	21.64	28.37	38.96
STD	7.39 ± 0.26	8.27 ± 0.13	8.41 ± 0.18 ∗	8.17 ± 0.32 ∗∗	7.19 ± 0.07 ∗∗∗	1.93	8.62	14.97	32.22

*Note:* Results were expressed as average ± SEM, whereas N = 4 and levels of significance were denoted as ∗p < 0.05, ∗∗p < 0.01, and ∗∗∗p < 0.001.

### 3.5. Hypoglycemic

The methanolic crude extract of *E. crassipes* flowers demonstrated dose‐dependent antihyperglycemic activity in the glucose tolerance test (Table 4). The control group showed a sharp glucose rise at 30 min (10.02 ± 0.55 mmol/L) before gradually declining. The STD drug significantly reduced glucose at 120 min (2.68 ± 0.58, ∗∗p < 0.01), showing 49.53% reduction. CME at 200 mg/kg lowered glucose steadily, reaching 3.77 ± 0.70 mmol/L at 120 min (28.77% reduction). The 400 mg/kg dose significantly suppressed glucose rise at 30 min (6.40 ± 1.28, ∗p < 0.05) and maintained stable levels thereafter, indicating strong glycemic control. The 600 mg/kg group also moderated glucose excursions, with 4.80 ± 0.16 mmol/L at 120 min (9.43% reduction). Overall, the methanolic extract of *E. crassipes* flowers produced significant and sustained antihyperglycemic effects, effectively reducing postprandial glucose excursions. The 400 and 600 mg/kg doses closely approached the efficacy of glibenclamide, with 400 mg/kg providing the most consistent glycemic regulation, suggesting its potential as an optimal therapeutic dose.

**Table 4:** Hypoglycemic activities of the methanolic crude extract of the flower of *E. crassipes* through glucose tolerance test in mice.

Groups	Average glucose level				% change in glucose level			
	0 min	30 min	60 min	120 min	0 min	30 min	60 min	120 min
Control	5.20 + 0.38	10.02 + 0.55	7.55 + 0.22	5.30 + 0.18				
STD	3.70 + 0.32∗	9.68 + 2.99	6.95 + 1.73	2.68 + 0.58∗∗	28.85	3.49	7.95	49.53
CME 200	4.50 + 0.44	7.53 + 1.76	6.90 + 0.77	3.77 + 0.70	13.46	24.94	8.61	28.77
CME 400	4.78 + 0.36	6.40 + 1.28∗	6.48 + 0.35∗	5.10 + 0.16	8.17	36.16	14.24	3.77
CME 600	4.76 + 0.39	8.40 + 1.90	7.08 + 0.88	4.80 + 0.16	8.17	16.21	6.29	9.43

*Note:* Results were expressed as average ± SEM, whereas N = 4 and levels of significance were denoted as ∗p < 0.05, ∗∗p < 0.01, and ∗∗∗p < 0.001.

### 3.6. Docking

The molecular docking study revealed notable binding affinities of the isolated compounds with anti‐inflammatory, cytotoxic, and hypoglycemic targets (Table 5). Among the isolated flavonoids, luteolin exhibited the strongest binding interactions across all targets, with values of −5.6 (NF‐*κ*B), −7.9 (TNF‐*α*), −8.9 (EGFR), −6.9 (BCL‐2), −9.6 (GLUT3), and −8.7 kcal/mol (*α*‐amylase). Apigenin and nortangeretin also demonstrated consistently high affinities, particularly in cytotoxic (−8.6 kcal/mol for both with EGFR) and hypoglycemic targets (−9.4 and −9.6 kcal/mol, respectively, with GLUT3). These findings highlight their potential as multitarget therapeutic agents.

**Table 5:** Molecular docking of isolated compounds from the DCM fraction of the methanolic extract of the flower of *E. crassipes* against different target receptors.

SL no	Pub Chem ID	Compound name	Anti‐inflammatory		Cytotoxic		Hypoglycemic	
			NF‐*κ*B (5LDE)	TNF‐*α* (2AZ5)	EGFR (1XKK)	BCL‐2 (2W3L)	GLUT3 (4ZWB)	*α*‐Amylase (1HNY)
1	5280443	4 ^′^,5,7‐Trihydroxyflavone (apigenin)	−5.7	−7.6	−8.6	−6.9	−9.4	−8.6
2	5280445	3 ^′^,4 ^′^,5,7‐Tetrahydroxyflavone (luteolin)	−5.6	−7.9	−8.9	−6.9	−9.6	−8.7
3	96506	5,6,7,8,4 ^′^‐Pentahydroxyflavone (nortangeretin)	−5.7	−7.8	−8.6	−7.1	−9.6	−8.3
4	78016	Methyl 3,5‐dihydroxy‐4‐methoxy benzoate	−4	−5.6	−5.5	−4.7	−6.4	−5.3
5	135	4‐Hydroxy benzoic acid	−3.9	−5.1	−6.1	−4.8	−6.2	−5.1
STD	2244	Aspirin	−4.5	−5.8	−6.1	−5	−7.1	−5.5
	5978	Vincristine			−6.6	−7.4		
	208908	Lapatinib			−10.7	−8.4		
	3488	Glibenclamide					−10.2	−8.8

In contrast, the simple phenolic derivatives showed comparatively weaker affinities. Methyl 3,5‐dihydroxy‐4‐methoxy benzoate displayed moderate interactions ranging from −4.0 to −6.4 kcal/mol across the targets, whereas 4‐hydroxybenzoic acid exhibited the weakest binding, with values between −3.9 and −6.2 kcal/mol.

Among the standard reference drugs, aspirin showed moderate activity with anti‐inflammatory proteins (−4.5 and −5.8 kcal/mol) and lower affinities against cytotoxic and hypoglycemic targets. Vincristine showed stronger cytotoxic interactions (−6.6 and −7.4 kcal/mol), whereas lapatinib demonstrated the highest affinity for EGFR (−10.7 kcal/mol) and BCL‐2 (−8.4 kcal/mol), validating the docking protocol. For hypoglycemic activity, glibenclamide exhibited the strongest interactions (−10.2 and −8.8 kcal/mol), serving as a positive control.

**Figure 7:**
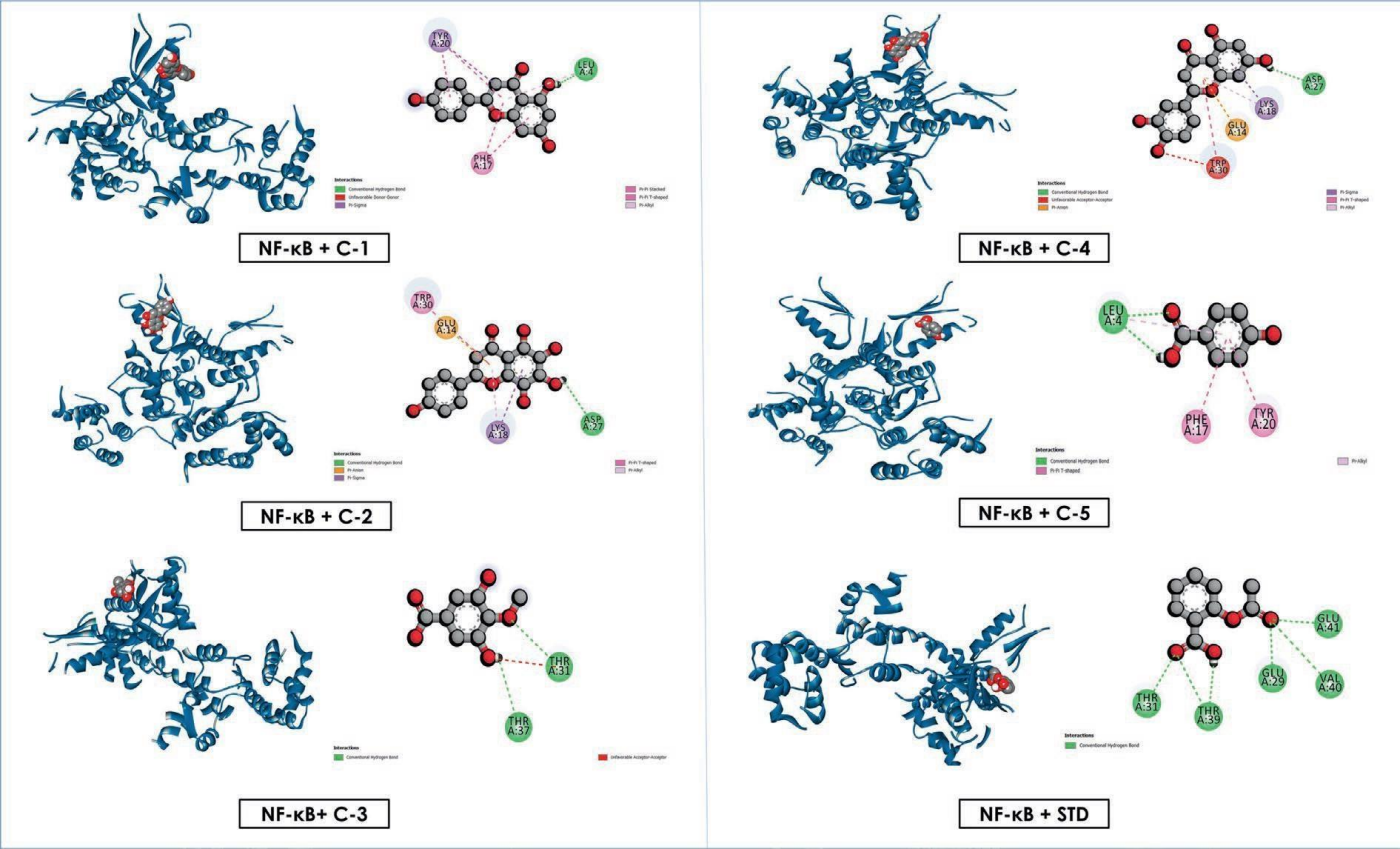
Graphical depiction of isolated compounds docked with NF‐*κ*B, represented through both 2D and 3D interaction maps.

**Figure 8:**
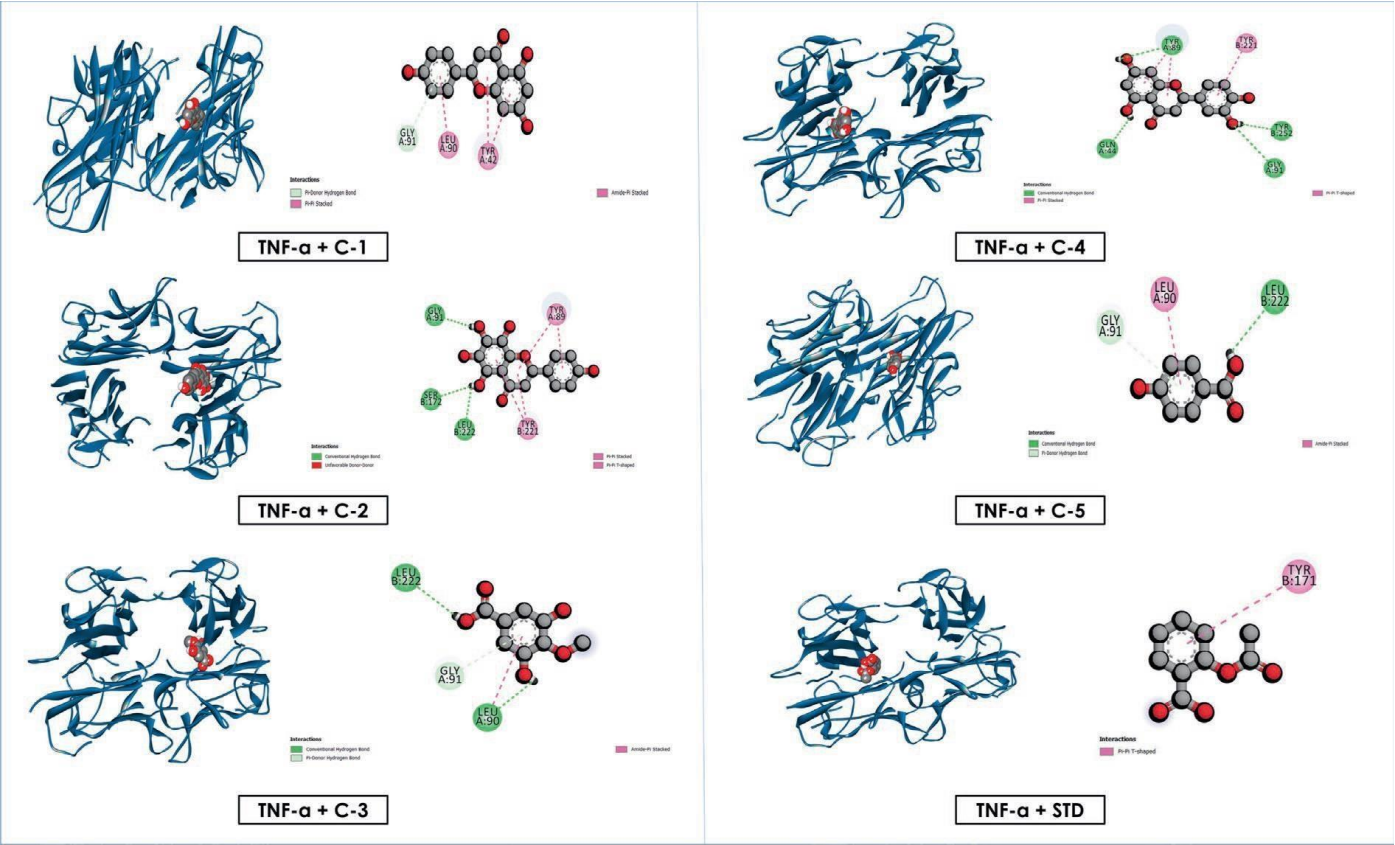
Graphical depiction of isolated compounds docked with TNF‐*α*, represented through both 2D and 3D interaction maps.

**Figure 9:**
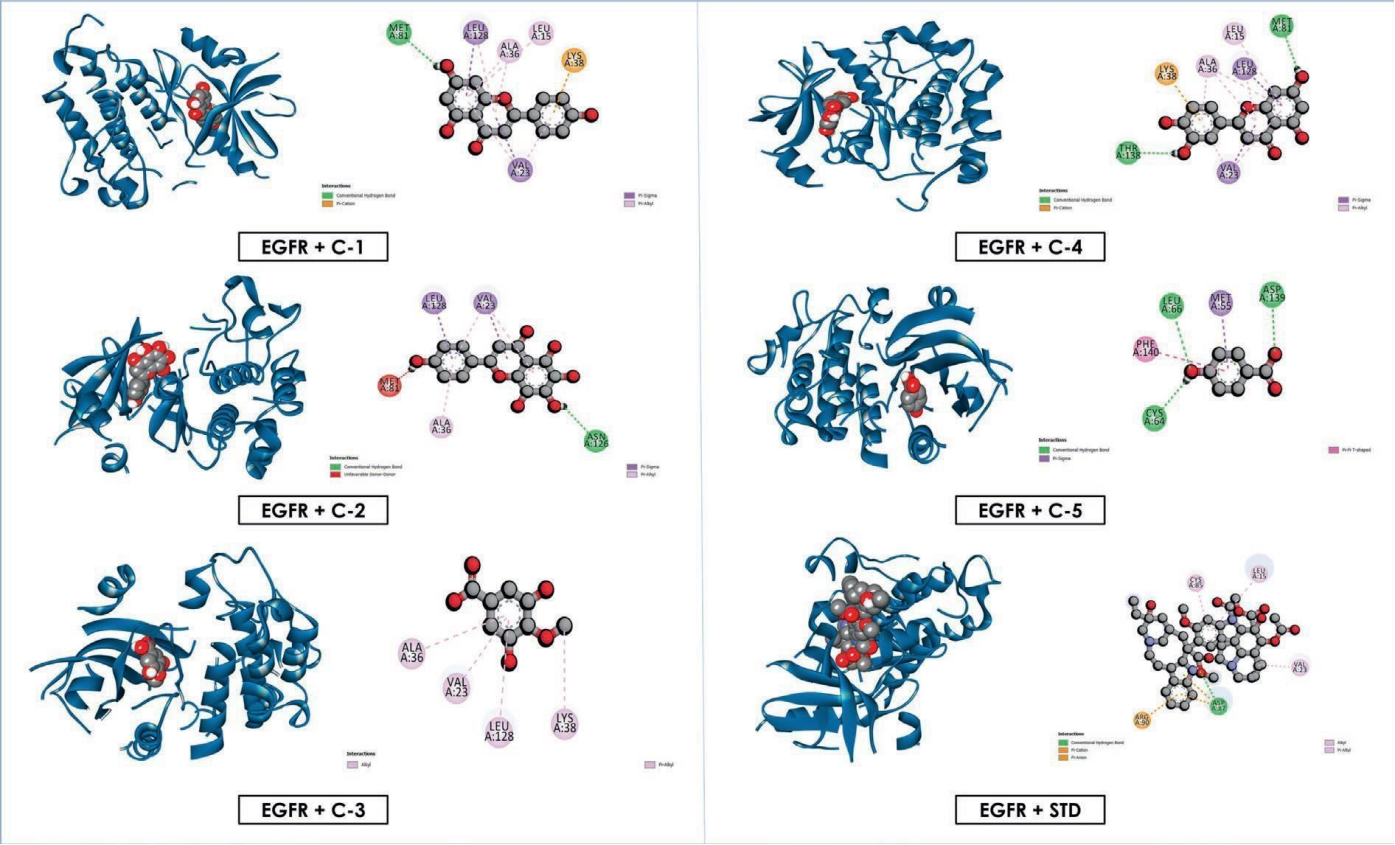
Graphical depiction of isolated compounds docked with EGFR, represented through both 2D and 3D interaction maps.

**Figure 10:**
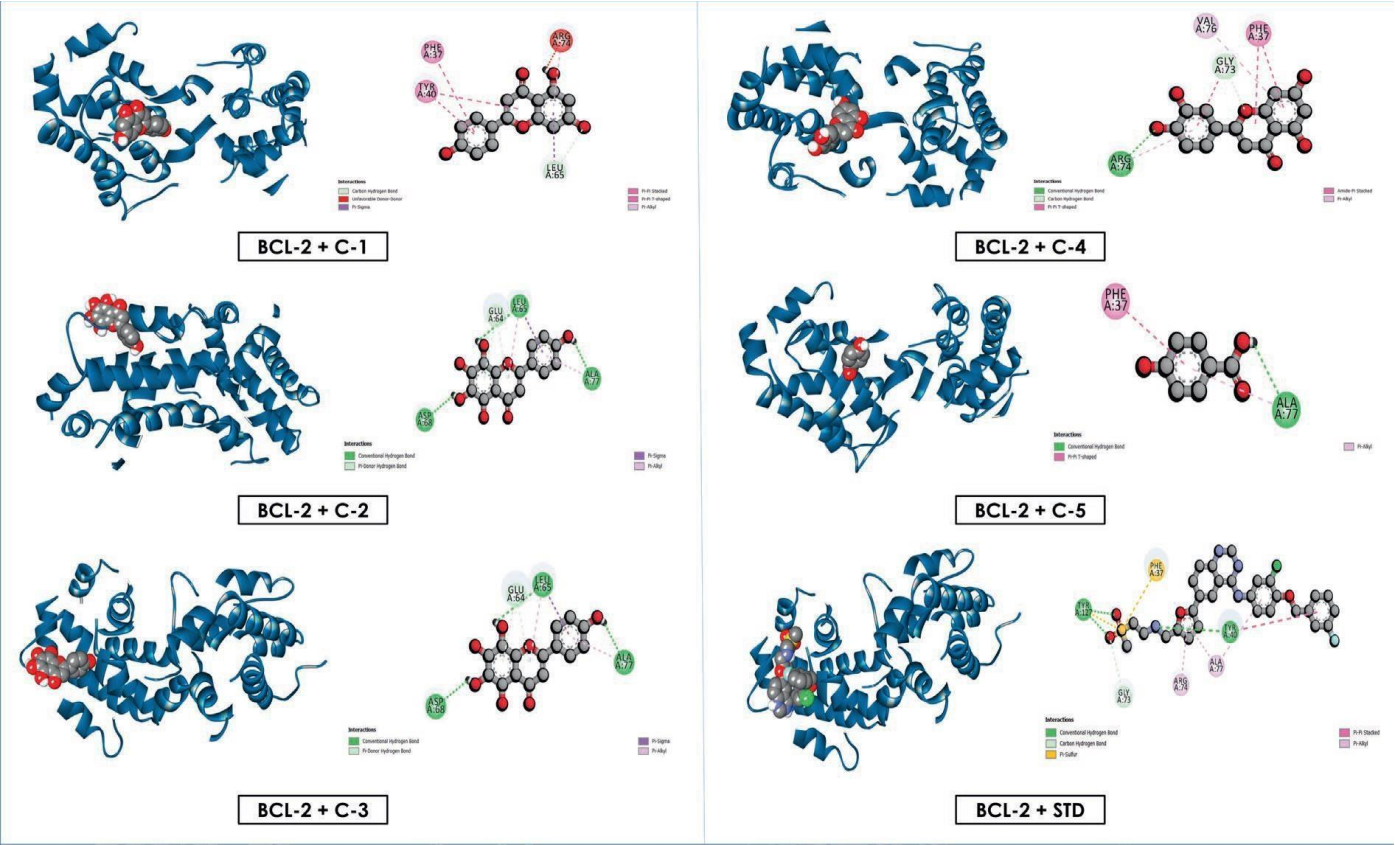
Graphical depiction of isolated compounds docked with BCL‐2, represented through both 2D and 3D interaction maps.

**Figure 11:**
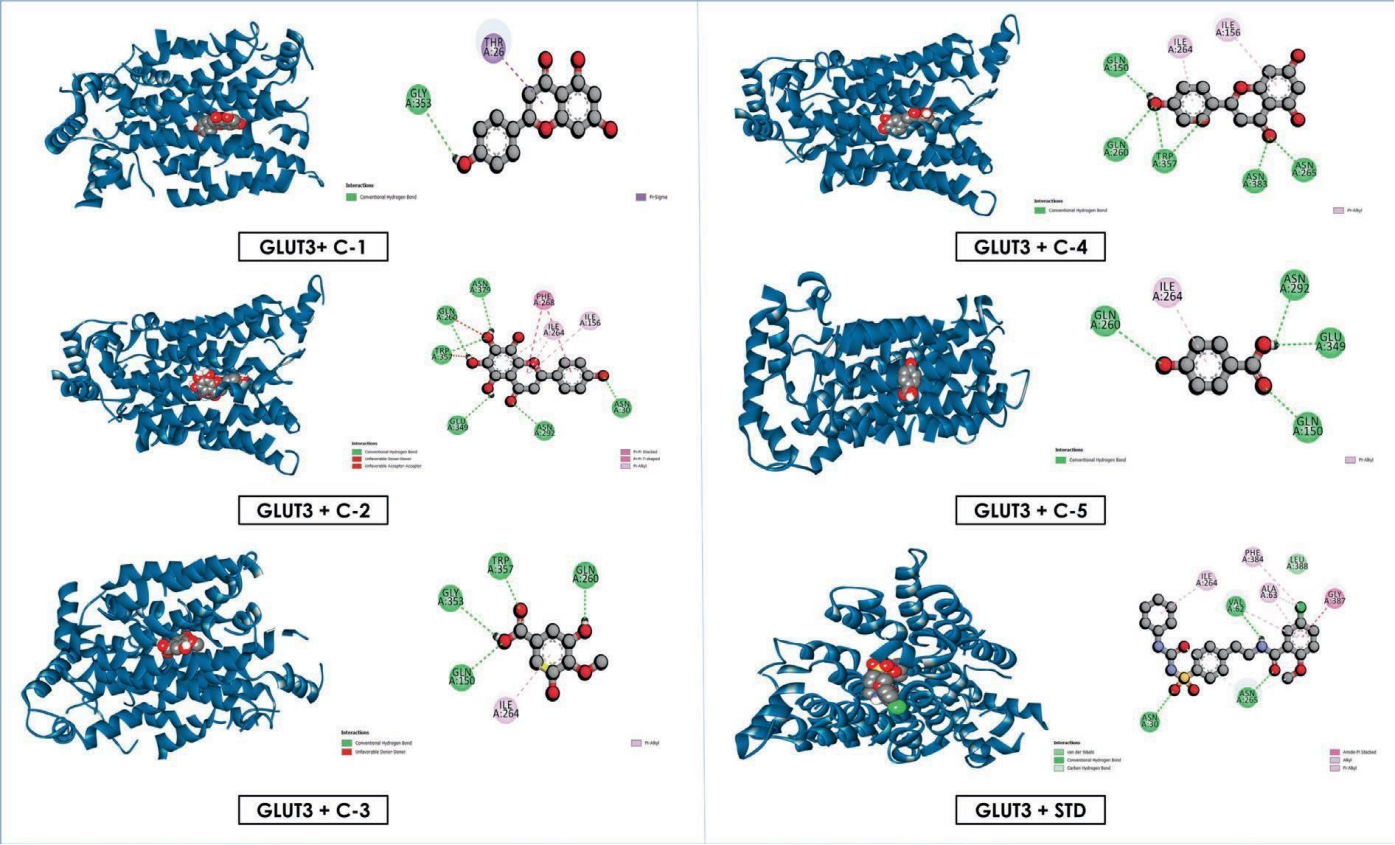
Graphical depiction of isolated compounds docked with GLUT3, represented through both 2D and 3D interaction maps.

**Figure 12:**
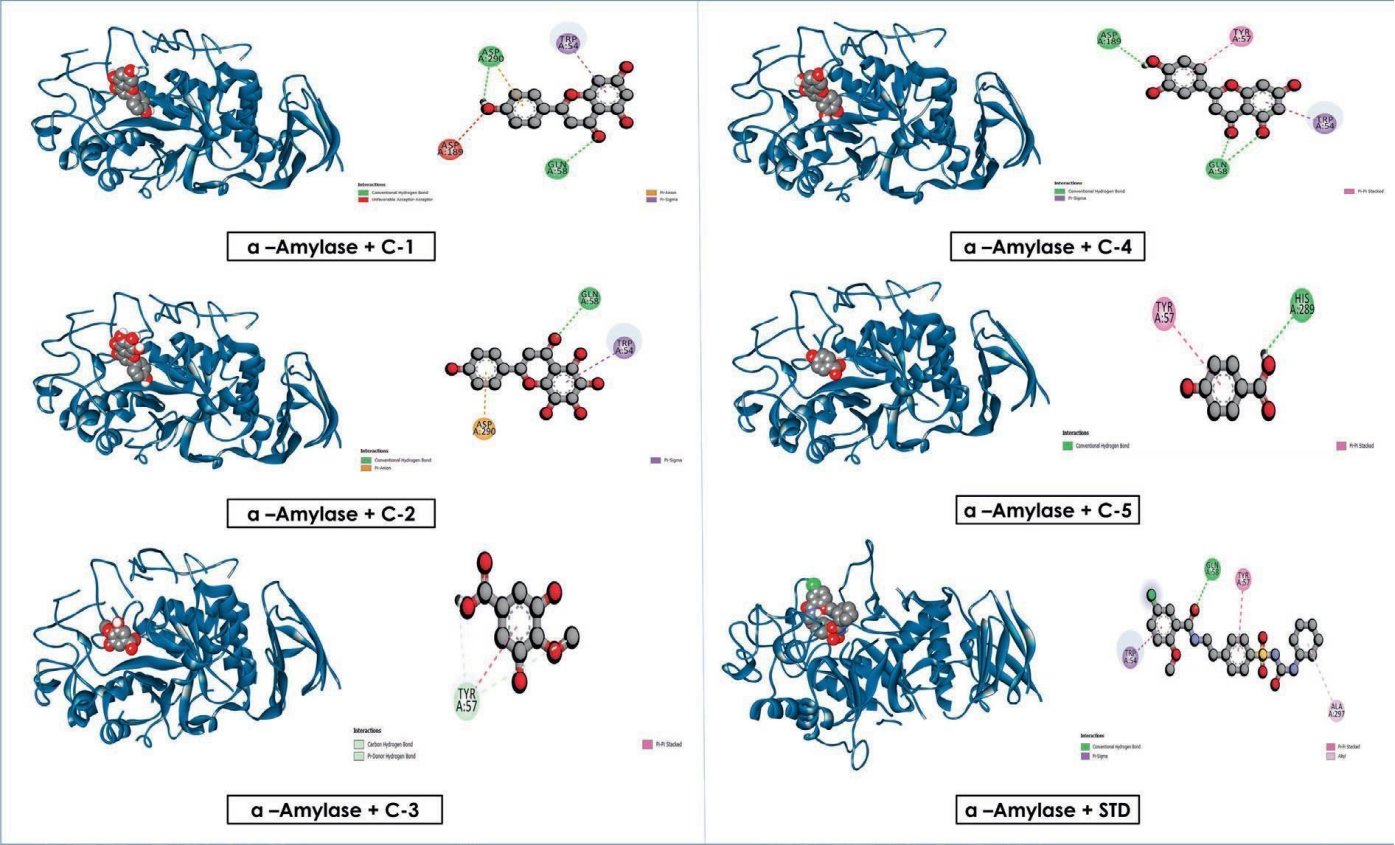
Graphical depiction of isolated compounds docked with *α*‐amylase, represented through both 2D and 3D interaction maps.

Overall, these results emphasize that luteolin, apigenin, and nortangeretin consistently demonstrated superior docking scores and stable binding orientations across inflammation‐, cancer‐, and diabetes‐related targets. Their interactions, validated by both docking scores and visualization (Figures 7, 8, 9, 10, 11, and 12), highlight their broad therapeutic potential, whereas the simpler phenolic compounds (methyl 3,5‐dihydroxy‐4‐methoxy benzoate and 4‐hydroxybenzoic acid) showed weaker and less specific interactions.

## 4. Discussion

This study presents the first report on compound isolation from the DSF the methanolic extract of *E. crassipes* flowers, confirmed through structural elucidation employing spectroscopic analyses. Previous studies on this plant have primarily concentrated on general phytochemical screening and profiling; however, systematic isolation and characterization of pure compounds from the floral extract have not been documented to date [10]. This novelty strengthens the phytopharmacological relevance of *E. crassipes* as a source of bioactive natural products with potential drug discovery implications. From the DSF of the methanolic extract, five distinct compounds were successfully isolated following consecutive chromatographic separation and purification. These include three flavonoids—4 ^′^,5,7‐trihydroxyflavone (apigenin), 3 ^′^,4 ^′^,5,7‐tetrahydroxyflavone (luteolin), and 5,6,7,8,4 ^′^‐pentahydroxyflavone (nortangeretin)—as well as two simple phenolic derivatives, namely methyl 3,5‐dihydroxy‐4‐methoxy benzoate and 4‐hydroxy benzoic acid. The successful isolation of these structurally diverse compounds underscores the polyphenolic richness of *E. crassipes* flowers. The predominance of flavonoids highlights the plant′s potential as a reservoir of multifunctional bioactive molecules, whereas the occurrence of phenolic acids and esters further supports its candidacy as a valuable source of lead compounds for therapeutic development.

The ^1^H NMR spectrum (MeOD, 600 MHz) of Compound 1 reveals several characteristic signals that confirm its flavonoid structure. The doublet at 7.86 ppm (*J* = 8.4 Hz) corresponds to the aromatic protons H‐2 ^′^ and H‐6 ^′^ on the B‐ring of the flavonoid. These protons are in an ortho relationship to each other, as indicated by the coupling constant of 8.4 Hz, which is typical for protons in this position. The electron‐withdrawing effect of the adjacent hydroxyl groups influences their chemical shift, causing them to resonate downfield. The doublet at 6.936 ppm (*J* = 8.4 Hz) is attributed to the aromatic protons H‐3 ^′^ and H‐5 ^′^ on the same B‐ring. These protons also exhibit an ortho relationship, as reflected by their similar coupling constant. Their position is further confirmed by the symmetry of the B‐ring, which results in two distinct sets of doublets corresponding to the four protons on this ring. The difference in chemical shift between these two sets of doublets is due to the electronic effects of the hydroxyl groups at C‐4 ^′^, which alter the electron density around these protons. The singlet at 6.602 ppm corresponds to H‐3, which is positioned on the central fused ring system of the flavonoid. This proton is strongly influenced by the extended conjugation in the molecule, leading to a notable downfield shift. The absence of coupling suggests that it is not adjacent to another proton, which aligns with its expected position in the structure. Another singlet at 6.466 ppm is assigned to H‐8, a proton located on the A‐ring of the flavonoid. The second singlet at 6.216 ppm corresponds to H‐6, which is also on the A‐ring. The fact that both protons appear at the same chemical shift suggests that they experience similar electronic environments, which is common in flavonoids with symmetrical substitution patterns. These protons are positioned between oxygenated carbons, leading to slight deshielding effects and a downfield chemical shift. Together, these signals confirm the presence of the hydroxylated flavonoid apigenin, characterized by a fully conjugated system spanning the three rings. The electron‐donating and electron‐withdrawing effects of the hydroxyl groups influence the proton environments, leading to the observed chemical shifts. The NMR data align with the expected molecular framework of 4 ^′^,5,7‐trihydroxyflavone or apigenin, providing strong evidence for its structure.

The ^1^H NMR spectrum (MeOD‐d₄, 600 MHz) of Compound 2 shows characteristic signals that confirm its flavonoid structure. The singlet at 6.210 ppm corresponds to the proton H‐6, which is located on the A‐ring of the flavonoid. The absence of splitting suggests that this proton has no neighboring protons, making it a distinctive feature of this position. Similarly, the singlet at 6.445 ppm is assigned to H‐8, another proton on the A‐ring, confirming the structure of this part of the molecule. The similarity in the chemical shifts of H‐6 and H‐8 is due to their positioning within the ring system, where they experience similar electronic effects from nearby oxygen atoms. The singlet at 6.545 ppm corresponds to H‐3, which is also located on the A‐ring. This proton resonates at a slightly downfield position due to its involvement in the extended conjugation system of the flavonoid, which influences its chemical environment. The lack of coupling further supports its isolated position within the ring system. The doublet at 6.905 ppm (*J* = 7.8 Hz) corresponds to H‐5 ^′^, which is located on the B‐ring of the flavonoid. The coupling constant of 7.8 Hz indicates an ortho relationship with an adjacent proton, reinforcing the expected connectivity of the ring. The doublet at 7.382 ppm (*J* = 9 Hz) is assigned to H‐2 ^′^ and H‐6 ^′^, which are also located on the B‐ring. The 9‐Hz coupling constant suggests an ortho arrangement between these two protons, confirming the substitution pattern of the B‐ring. Together, these NMR signals confirm the structure of luteolin, a flavonoid featuring a hydroxylated B‐ring and an extended conjugated system that influences the chemical shifts of its aromatic protons. The observed pattern of singlets and doublets aligns with the expected proton environments in the structure of 3 ^′^,4 ^′^,5,7‐tetrahydroxyflavone or luteolin.

The observed NMR data strongly support the presence of a 5,6,7,8,4 ^′^‐pentahydroxyflavone skeleton. The doublets at *δ* 7.346 and *δ* 6.891 ppm with identical coupling constants (*J* = 8.4 Hz) confirm an ortho‐coupled pair of protons on the B‐ring, consistent with protons at H‐5 ^′^ and H‐6 ^′^ in a 1,4‐disubstituted pattern. The singlets at *δ* 7.431 and *δ* 7.083 are likely from additional aromatic protons at positions with no neighboring protons, or possibly arise from minor noninteracting environments within the B‐ring. The absence of A‐ring aromatic proton signals suggests full hydroxylation at Positions 5, 6, 7, and 8, which is a defining feature of the compound. The singlet at *δ* 12.564 ppm is diagnostic of a chelated hydroxyl group at Position 5, which forms a strong intramolecular hydrogen bond with the adjacent carbonyl group at Position 4. This is commonly observed in flavonoids and contributes to the stability of the flavanone core. The aliphatic signals confirm the flavanone structure, with a typical downfield H‐2 proton coupling with the two diastereotopic H‐3 methylene protons. Their respective coupling constants and chemical shifts match literature‐reported values for flavanones, further supporting the structural assignment. Taken together, the spectrum provides a clear confirmation that the isolated compound is consistent with the structure of 5,6,7,8,4 ^′^‐pentahydroxyflavone or nortangeretin, in agreement with reference data such as Sharmin et al. (2016).

The 1H NMR spectrum (CDCl3, 600 MHz) of Compound 4 provides distinct signals that confirm its structure. The doublet at 7.225 ppm, which appears as a singlet for two protons, is assigned to the aromatic protons at positions H‐2 and H‐6 on the benzene ring. These two protons are in an equivalent, symmetrical environment due to the presence of the hydroxyl and methoxy groups at the 3 and 4 positions, respectively, resulting in identical chemical shifts for these protons. The singlet at 3.865 ppm corresponds to the three protons of the methoxy group attached to the ester functionality (–OCOCH3) at Position 4 of the benzene ring. This signal indicates the presence of the ester group (–COOCH3), which is typical for methyl esters. Another singlet at 3.892 ppm is assigned to the methoxy group (–OCH3) attached to the benzene ring at Position 4, confirming the presence of this functional group. Together, the singlets for both methoxy groups and the symmetric aromatic proton signals suggest that the molecule has a symmetrical substitution pattern on the benzene ring. The positions of the hydroxyl and methoxy groups further support the structure of methyl 3,5‐dihydroxy‐4‐methoxybenzoate. The proton environments observed in the NMR spectrum align with the expected functional groups, confirming the identity of the compound.

The 1H NMR spectrum (CDCl3, 600 MHz) of Compound 5 shows characteristic signals that help confirm its structure. The doublet at 7.99 ppm (*J* = 7.8 Hz) corresponds to the two protons at positions H‐2 and H‐3 on the benzene ring. These protons are in an ortho‐coupling relationship with each other, as indicated by the coupling constant of 7.8 Hz, suggesting that they are adjacent to one another on the ring. Similarly, the doublet at 6.88 ppm (*J* = 8.4 Hz) is attributed to the two protons at positions H‐3 and H‐5. These protons are also in an ortho‐coupling relationship, as shown by the coupling constant of 8.4 Hz, and are positioned next to each other in the ring. The overall splitting pattern with two doublets, each corresponding to adjacent aromatic protons, supports the typical structure of 4‐hydroxybenzoic acid, with a hydroxyl group at Position 4 on the benzene ring. The 1H NMR spectrum confirms the expected substitution pattern and provides clear evidence for the presence of the hydroxyl group at Position 4, as well as the relative positions of the other protons on the benzene ring.

The brine shrimp lethality assay demonstrated that the CME and DSF exerted the strongest cytotoxic activity, as evidenced by their relatively low LC₅₀ values. This activity is likely attributable to the presence of secondary metabolites such as alkaloids, flavonoids, or terpenoids, which are known to interfere with cellular viability and proliferation [42, 66]. The petroleum‐soluble fraction (PSF) showed moderate activity, whereas the ESF and ASF were less potent, as indicated by their higher LC₅₀ values. These differences suggest that the most cytotoxic phytoconstituents are enriched in CME and DSF. Importantly, the relatively high LC₅₀ of ASF also suggests low toxicity, which may be advantageous in contexts where safety is prioritized. These preliminary results warrant further studies using cancer cell lines to validate the anticancer potential of CME and DSF.

The erythrocyte membrane stabilization assay revealed that DSF and ASF provided the highest inhibition of hemolysis, particularly under heat/cold‐induced stress, where their effects (80.66% and 79.11%, respectively) approached that of diclofenac sodium (87.57%). This strong activity suggests the presence of flavonoids, phenolics, and tannins that stabilize cell membranes and inhibit protein denaturation, mechanisms commonly linked to anti‐inflammatory actions [10, 67].

The extract demonstrated greater efficacy in the heat‐ and cold‐induced hemolysis models compared to the hypotonic model, suggesting that the bioactive constituents of *E. crassipes* may be particularly effective under conditions associated with thermal or oxidative stress, which are commonly involved in inflammatory processes [27]. Furthermore, in vivo anti‐inflammatory evaluation confirmed a time‐ and dose‐dependent response. Higher doses (400–600 mg/kg) produced sustained inhibition of inflammation, with effects at later time points surpassing those of the STD drug. This delayed but stronger response may indicate that the extract acts through pathways that become prominent in the chronic phase of inflammation, such as suppression of prostaglandin biosynthesis or downregulation of pro‐inflammatory cytokines [68]. Such sustained activity distinguishes *E. crassipes* from conventional NSAIDs and suggests potential use in long‐term inflammatory conditions.

In the oral glucose tolerance test, *E. crassipes* flower extract significantly attenuated postprandial hyperglycemia, with the 400 mg/kg dose showing the most consistent effect. This finding indicates an optimal concentration of active compounds for glycemic regulation. Phytochemical constituents of *E. crassipes*, including flavonoids, alkaloids, tannins, and phenolic acids, are well‐documented for their roles in stimulating insulin secretion, enhancing glucose uptake, and modulating intestinal glucose absorption [10, 69]. Interestingly, while the 600 mg/kg dose provided stable glucose control, it did not show greater efficacy than the 400 mg/kg dose, suggesting a possible saturation effect or complex dose–response dynamics, as reported in other phytopharmacological studies [70]. Although glibenclamide showed a stronger and more rapid effect, the extract′s gradual and sustained glucose‐lowering action suggests a multifactorial mechanism, likely involving antioxidant, anti‐inflammatory, and metabolic modulation rather than a direct insulinotropic effect [71]. These findings validate the ethnomedicinal use of *E. crassipes* in managing diabetes and highlight its potential as a complementary antidiabetic therapy.

Taken together, the findings of this study suggest that *E. crassipes* flower extracts possess multidimensional pharmacological activities: cytotoxic (CME and DSF), anti‐inflammatory (DSF and ASF), membrane stabilizing (DSF and ASF), and antihyperglycemic (400 mg/kg extract). The consistency of DSF′s performance across assays suggests that it may contain a higher concentration of key bioactive metabolites, deserving priority in bioassay‐guided fractionation. The alignment of these results with prior phytopharmacological studies of Bangladeshi medicinal plants [69] strengthens the case for exploring *E. crassipes* as a source of novel therapeutic agents. Importantly, while the cytotoxic activity highlights its anticancer potential, the anti‐inflammatory and antihyperglycemic activities point to broader clinical applications. As a result, the present study combined molecular docking, ADMET profiling, physicochemical radar analysis, and the BOILED‐Egg predictive model to evaluate the pharmacological potential of five isolate compounds: apigenin, luteolin, nortangeretin, methyl 3,5‐dihydroxy‐4‐methoxy benzoate, and 4‐hydroxy benzoic acid. Together, these in silico approaches provide a comprehensive assessment of drug‐likeness and therapeutic promise across multiple disease domains.

Docking studies revealed that luteolin, apigenin, and nortangeretin exhibited consistently strong binding affinities across anti‐inflammatory, cytotoxic, and hypoglycemic targets, highlighting their multitarget potential. Luteolin was the most potent, with affinities of −8.9 kcal/mol against the cytotoxic receptor 1XKK and −9.6 kcal/mol against the hypoglycemic target 4ZWB. These results are consistent with experimental studies showing luteolin′s ability to inhibit PI3K/Akt and NF‐*κ*B pathways, thereby suppressing tumor growth and inflammation, while also enhancing insulin sensitivity through AMPK activation [72–74]. Apigenin also showed notable binding strength, particularly −8.6 kcal/mol with cytotoxic and hypoglycemic proteins, which aligns with its known ability to induce apoptosis, regulate the cell cycle, and reduce inflammatory mediators via suppression of COX‐2 and iNOS expression [75, 76]. Nortangeretin demonstrated comparable affinities, especially against the anti‐inflammatory protein 2az5 (−7.8 kcal/mol) and hypoglycemic receptor 4ZWB (−9.6 kcal/mol), corroborating reports that flavones modulate cytokine signaling and glucose metabolism via AMPK‐related mechanisms [7]. By contrast, methyl 3,5‐dihydroxy‐4‐methoxy benzoate and 4‐hydroxy benzoic acid had weaker docking scores (−3.9 to −6.4 kcal/mol), reflecting their less complex molecular scaffolds and reduced interaction potential.

Computational ADMET profiling has become an established early‐stage screening strategy in modern drug discovery, enabling prediction of oral bioavailability, toxicity, and pharmacokinetic behavior prior to experimental validation [77–79]. Consistent with these principles, the present ADMET analysis indicated favorable pharmacokinetic properties and acceptable safety profiles for the isolated flavonoid compounds, as summarized in Table 6. All major candidates displayed high intestinal absorption (> 80%), compliance with Lipinski′s Rule of Five, and moderate bioavailability scores. However, poor water solubility and predicted recognition as P‐glycoprotein substrates may limit oral bioavailability, a challenge frequently observed in polyphenolic compounds [80]. Luteolin and nortangeretin were predicted to have high distribution volumes, suggesting efficient tissue penetration. Importantly, none of the flavonoids were predicted to be substrates of CYP3A4, reducing the likelihood of extensive first‐pass metabolism. Selective CYP inhibition, such as CYP1A2 inhibition by luteolin and apigenin, indicates possible drug–drug interactions that warrant further evaluation. Toxicity predictions were favorable across all compounds, with negative AMES mutagenicity, no hepatotoxicity, and no risk of cardiotoxicity via hERG inhibition, suggesting a wide safety margin for potential therapeutic use.

**Table 6:** ADME/T of isolated compounds from the DCM fraction of the methanolic extract of the flower of *E. crassipes.*

Properties	Model name (unit)	4,5,7‐Trihydroxyflavone (apigenin)	3 ^′^,4,5,7‐Tetrahydroxyflavone (luteolin)	5,6,7,8,4 ^′^‐Pentahydroxyflavone (nortangeretin)	Methyl 3,5‐dihydroxy‐4‐methoxy benzoate	4‐Hydroxy benzoic acid
Absorption	Water solubility (log mol/L)	−3.329	−3.094	−2.922	−2.138	−1.877
	Caco2 permeability (log Papp in 10^−6^ cm/s)	1.007	0.096	−0.576	0.255	1.151
	Intestinal absorption (human) (% absorbed)	93.25	81.13	60.148	85.695	83.961
	Skin permeability (log Kp)	−2.735	−2.735	−2.735	−2.735	−2.723
	P‐glycoprotein substrate	Yes	Yes	Yes	Yes	No
	P‐glycoprotein I inhibitor	No	No	No	No	No
	P‐glycoprotein II inhibitor	No	No	No	No	No
Distribution	VDss (human) (log L/kg)	0.822	1.153	1.73	−1.397	−1.557
	Fraction unbound (human) (Fu)	0.147	0.168	0.214	0.638	0.592
	BBB permeability	−0.734	−0.907	−1.409	−0.826	−0.334
	CNS permeability	−2.061	−2.251	−3.009	−2.773	−3.21
Metabolism	CYP2D6 substrate	No	No	No	No	No
	CYP3A4 substrate	No	No	No	No	No
	CYP1A2 inhibitor	Yes	Yes	Yes	No	No
	CYP2C19 inhibitor	Yes	No	No	No	No
	CYP2C9 inhibitor	No	Yes	No	No	No
	CYP2D6 inhibitor	No	No	No	No	No
	CYP3A4 inhibitor	No	No	No	No	No
Excretion	Total clearance (log mL/min/kg)	0.566	0.495	0.368	0.596	0.593
	Renal OCT2 substrate	No	No	No	No	No
Toxicity	AMES toxicity	No	No	No	No	No
	Max. tolerated dose (human) (log mg/kg/day)	0.328	0.499	0.501	1.331	0.846
	hERG I inhibitor	No	No	No	No	No
	hERG II inhibitor	No	No	No	No	No
	Oral rat acute toxicity (LD50) (mol/kg)	2.45	2.455	2.475	2.321	2.255
	Oral rat chronic toxicity (LOAEL) (log mg/kg_bw/day)	2.298	2.409	3.339	2.856	2.483
	Hepatotoxicity	No	No	No	No	No
	Skin sensitization	No	No	No	No	No
	*Tetrahymena pyriformis* toxicity (log *μ*g/L)	0.38	0.326	0.285	0.282	0.268
	Minnow toxicity (log mM)	2.432	3.169	4.004	2.663	1.812
Drug‐likeness	Lipinski′s RO5 violation	Yes; 0 violation	Yes; 0 violation	Yes; 0 violation	Yes; 0 violation	Yes; 0 violation
	Bioavailability score (%)	0.55	0.55	0.55	0.56	0.85

**Figure 13:**
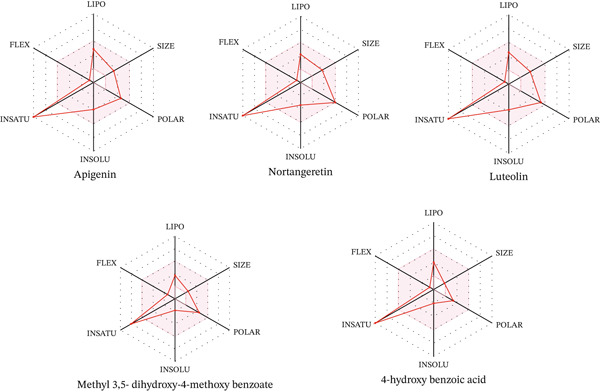
Radar images of isolated from the DCM fraction of the methanolic extract of the flower of *E. crassipes.*

Radar plot analysis further highlighted the favorable drug‐likeness characteristics of luteolin, apigenin, and naringenin, which exhibited well‐balanced physicochemical profiles in terms of lipophilicity, polarity, molecular size, and aqueous solubility (Figure 13). Such balanced distributions are widely recognized as key determinants for oral bioavailability and pharmacokinetic suitability of small‐molecule candidates [77, 81]. The pronounced degree of molecular unsaturation observed in these flavonoids, arising from their aromatic and conjugated ring systems, confers structural advantages for effective *π*–*π* stacking and hydrogen‐bonding interactions with biological macromolecules, thereby enhancing binding stability and docking performance [73, 80]. In contrast, comparatively simpler phenolic constituents displayed reduced radar plot complexity, which correlated with their lower binding affinities and limited interaction potential within the active site.

**Figure 14:**
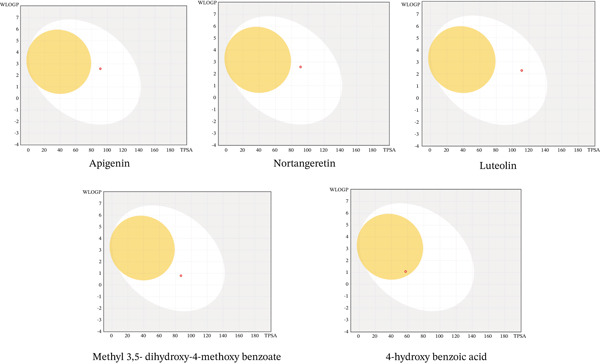
BOILED‐Egg images of isolated from the DCM fraction of the methanolic extract of the flower of *E. crassipes.*

The BOILED‐Egg predictive model was employed to further evaluate the gastrointestinal absorption and blood–brain barrier (BBB) permeability of the selected compounds (Figure 14). The analyzed flavonoids were predominantly positioned within the white region of the model, indicating a high probability of passive gastrointestinal absorption, which aligns well with the ADMET‐based pharmacokinetic predictions [81, 82]. Notably, none of the flavonoids localized within the yellow region, suggesting limited BBB permeability. This pharmacokinetic profile supports their potential applicability in systemic conditions such as metabolic dysfunction, inflammatory disorders, and cancer, rather than in central nervous system–targeted therapies [77, 83]. In contrast, 4‐hydroxybenzoic acid exhibited a marginal tendency toward BBB penetration; however, its comparatively weak molecular docking interactions substantially limit its therapeutic significance despite favorable permeability characteristics.

Taken together, these findings identify luteolin, apigenin, and nortangeretin as promising multitarget drug candidates. Their ability to interact strongly with diverse protein targets, combined with favorable pharmacokinetic and safety profiles, supports their therapeutic potential in managing complex diseases such as diabetes, cancer, and chronic inflammation. Compared to STD drugs such as lapatinib (−10.7 kcal/mol, cytotoxic) and glibenclamide (−10.2 kcal/mol, hypoglycemic), which serve as strong single‐target controls, the flavonoids demonstrated the advantage of polypharmacology by engaging multiple pathways simultaneously. This attribute is particularly valuable for multifactorial conditions where single‐target therapies are often insufficient. Nevertheless, limitations related to solubility and efflux susceptibility highlight the need for formulation improvements, such as nanoparticle delivery or prodrug strategies. Future in vitro and in vivo experiments, as well as structural modifications to enhance solubility and metabolic stability, are necessary to translate these promising in silico findings into clinical potential.

## 5. Limitations and Future Perspectives

Although this study successfully reports for the first time the isolation and structure elucidation of pure bioactive flavonoids from *E. crassipes* flowers, it has several limitations. The work was primarily confined to phytochemical isolation and in silico molecular docking analysis, without extending to in vitro or in vivo biological validation. The pharmacological activities were predicted based on docking scores and ADMET profiling, which, while informative, cannot fully replicate the complexity of biological systems. Furthermore, only the DCM fraction of the methanolic extract was explored, leaving the possibility that other solvent fractions may harbor additional novel or potent compounds. Another limitation lies in the scope of docking studies, which were restricted to selected protein targets and hence do not capture the full therapeutic landscape of the isolated metabolites.

Looking forward, future research should focus on experimental validation of the pharmacological activities of these compounds through in vitro assays (e.g., cytotoxicity, anti‐inflammatory, and antidiabetic tests) followed by in vivo studies to confirm therapeutic efficacy and safety. Advanced techniques such as metabolomics, transcriptomics, and network pharmacology could provide deeper insight into the multitarget actions of these phytochemicals. Additionally, structural optimization and formulation studies could improve solubility, stability, and bioavailability, facilitating the transition from bench to bedside. Given the invasive nature and wide availability of *E. crassipes*, these findings open avenues for sustainable utilization of this plant as a source of lead molecules in drug discovery, turning an environmental nuisance into a pharmacological resource.

## 6. Conclusion

The present study provides the first evidence of pure compound isolation from the DSF of *E. crassipes* flowers, yielding three flavonoids (apigenin, luteolin, and nortangeretin) and two phenolic derivatives. These compounds, particularly the flavonoids, exhibited strong biological activity across cytotoxic, anti‐inflammatory, membrane stabilizing, and hypoglycemic assays. In silico docking and ADMET predictions reinforced these findings by highlighting their multitarget potential, favorable pharmacokinetics, and drug‐likeness. Collectively, the results suggest that *E. crassipes* flowers represent a promising source of bioactive molecules with therapeutic potential against cancer, diabetes, and inflammation‐related disorders. Further studies on bioassay‐guided fractionation, mechanistic validation, and formulation strategies are warranted to fully explore their clinical applicability.

**Nomenclature**
ADME/T: absorption, distribution, metabolism, excretion/toxicity
ADMET: absorption, distribution, metabolism, excretion, and toxicity
AMES: Ames mutagenicity test
AMP: adenosine monophosphate
AMPK: AMP‐activated protein kinase
ANOVA: analysis of variance
ASF: aqueous soluble fraction
BACE: beta‐site amyloid precursor protein cleaving enzyme
BBB: blood–brain barrier
BCL: B‐cell lymphoma protein
BMC: bone marrow cells
BOILED: Brain Or IntestinaL Estimate D permeation method (BOILED‐Egg model)
CME: crude methanolic extract
CNS: central nervous system
COX: cyclooxygenase
CYP: cytochrome P450
DACB: Directorate of Animal Care and Breeding
DCM: dichloromethane
DM: diabetes mellitus
DSF: dichloromethane soluble fraction
EA: ethyl acetate
EASF: ethyl acetate soluble fraction
EDTA: ethylenediaminetetraacetic acid
EGFR: epidermal growth factor receptor
ERC: Ethics Review Committee
ESF: ethyl soluble fraction
FELASA: Federation of European Laboratory Animal Science Associations
FTIR: Fourier‐transform infrared spectroscopy
GC: gas chromatography
GPC/SEC: gel permeation chromatography/size exclusion chromatography
ICDDR,B: International Centre for Diarrhoeal Disease Research, Bangladesh
LDL: low‐density lipoprotein
LH: luteinizing hormone
MS: mass spectrometry
MS/MS: tandem mass spectrometry
NF: nuclear factor
NMR: nuclear magnetic resonance
OD: optical density
PASS: prediction of activity spectra for substances
PDB: Protein Data Bank
PSF: petroleum ether soluble fraction
PET: petroleum ether
PTLC: preparative thin‐layer chromatography
RCSB: Research Collaboratory for Structural Bioinformatics
Rf: retention factor
RNA: ribonucleic acid
SEM: standard error of mean
STD: standard
TMS: tetramethylsilane
TNF: tumor necrosis factor
UV: ultraviolet
WHO: World Health Organization

## Author Contributions

**Hasin Hasnat:** conceptualization, methodology, investigation, formal analysis, data curation, software, writing – original draft, writing – review and editing; **Md. Mirazul Islam:** supervision, conceptualization, methodology, data curation, validation, writing – review and editing, project administration; **Suriya Akter Shompa:** investigation, formal analysis, data curation, software, writing – original draft, writing – review and editing; **Saima Jahan Riti:** investigation, formal analysis, writing – review and editing; **Safaet Alam:** resources, methodology, data curation, validation, visualization, writing – original draft; **Tanoy Saha:** supervision, methodology, data curation, visualization, writing – review and editing; **Mohammad Abdur Rashid:** supervision, conceptualization, validation, visualization, project administration, writing – review and editing.

## Funding

No funding was received for this manuscript.

## Conflicts of Interest

The authors declare no conflicts of interest.

## Supporting information


**Supporting Information** Additional supporting information can be found online in the Supporting Information section. Meta data of in vitro and in vivo assays.

## Data Availability

The data that support the findings of this study are available in the Supporting Information.
